# A Theory of Cheap Control in Embodied Systems

**DOI:** 10.1371/journal.pcbi.1004427

**Published:** 2015-09-01

**Authors:** Guido Montúfar, Keyan Ghazi-Zahedi, Nihat Ay

**Affiliations:** 1 Max Planck Institute for Mathematics in the Sciences, 04103 Leipzig, Germany; 2 Department of Mathematics and Computer Science, Leipzig University, 04009 Leipzig, Germany; 3 Santa Fe Institute, Santa Fe, New Mexico, United States of America; University of Vermont, UNITED STATES

## Abstract

We present a framework for designing cheap control architectures of embodied agents. Our derivation is guided by the classical problem of universal approximation, whereby we explore the possibility of exploiting the agent’s embodiment for a new and more efficient universal approximation of behaviors generated by sensorimotor control. This embodied universal approximation is compared with the classical non-embodied universal approximation. To exemplify our approach, we present a detailed quantitative case study for policy models defined in terms of conditional restricted Boltzmann machines. In contrast to non-embodied universal approximation, which requires an exponential number of parameters, in the embodied setting we are able to generate all possible behaviors with a drastically smaller model, thus obtaining cheap universal approximation. We test and corroborate the theory experimentally with a six-legged walking machine. The experiments indicate that the controller complexity predicted by our theory is close to the minimal sufficient value, which means that the theory has direct practical implications.

## Author Summary

Given a body and an environment, what is the brain complexity needed in order to generate a desired set of behaviors? The general understanding is that the physical properties of the body and the environment correlate with the required brain complexity. More precisely, it has been pointed that naturally evolved intelligent systems tend to exploit their embodiment constraints and that this allows them to express complex behaviors with relatively concise brains. Although this principle of parsimonious control has been formulated quite some time ago, only recently one has begun to develop the formalism that is required for making quantitative statements on the sufficient brain complexity given embodiment constraints. In this work we propose a precise mathematical approach that links the physical and behavioral constraints of an agent to the required controller complexity. As controller architecture we choose a well-known artificial neural network, the conditional restricted Boltzmann machine, and define its complexity as the number of hidden units. We conduct experiments with a virtual six-legged walking creature, which provide evidence for the accuracy of the theoretical predictions.

## Introduction

The goal of this article is to provide a framework that allows us to determine the complexity of a control architecture in accordance with the *cheap design* principle from embodied artificial intelligence [1, 2]. Cheap design in this context refers to the relatively low complexity of the brain or controller in comparison with the complexity of an observed behavior. A classical example is given by the Braitenberg vehicles [3], which are *Gedankenexperiments* designed to show how a seemingly complex behavior can result from very simple control structures. Braitenberg discusses several artificial creatures with simple wirings between sensors and actuators. He then describes how these systems produce a behavior that an external observer would classify as complex if the internal wirings were not revealed. Most interestingly, he then relates the wiring of his vehicles to various neural structures in the human brain. The idea of a simple wiring that leads to complex behaviors is also discussed in [1, 2], who present the walking behavior of an ant as an example. Without taking the embodiment and, in particular, the sensorimotor loop into account, the complex behavior (of a complex morphology) seems to require a complex control structure [2]. A strong indication that cheap design is a common principle in biological systems is given by the fact that the human brain accounts for only 2% of the body mass but is responsible for 20% of the entire energy consumption [4], which is also remarkably constant [5]. Further support for cheap design as a common principle is given by a recent study on the brain sizes of migrating birds. It is known that migrating birds have a reduced brain size compared with their resident relatives. Sol et al. [6] have studied various species and the affected brain regions and point out that the reduced brain sizes could be a direct result from the need to reduce energetic, metabolic and cognitive costs for migrating birds.

It is generally believed that cheap design of control architectures is possible whenever the morphology of the system can contribute to the control of behaviors, which is referred to as *morphological computation* [7, 8]. This kind of computation can be illustrated in the context of the human walking behavior, which only needs to be actively controlled during the stance phase. The swing phase results mainly from the interaction of the physical properties of the leg with the environment (gravity). This is demonstrated by the Passive Dynamic Walker [9], which is a purely mechanical system that resembles the physical properties of human legs. The human walking behavior is emulated as a result of the interaction of the mechanical system with its environment (gravity and a slope). It is an extreme example of cheap design that requires no active control at all.

Morphological computation has been identified as a prime concept within the field of embodied cognition about two decades ago [7]. However, only recently one has begun to develop a theoretical understanding of this concept [10–15]. Currently, the field does not provide sufficiently conclusive definitions that would allow us to analytically reveal the design principles of cheap control based on high values of morphological computation. Experimental evidence of such a coupling has been provided in evolutionary settings. For instance, the work of Auerbach and Bongard [16] shows that complex environments increase the selection pressure for complex morphologies, given a low-complexity controller. This suggests an evolutionary coupling between morphological computation and cheap design.

We are interested in quantifying to what extent a control structure can be reduced if the physical constraints are taken into account. Above, we referred to a system as cheaply designed, if it has a control structure of low complexity that produces behaviors which an external observer would classify as complex. In this work, we are not concerned with the complexity of the behavior. Instead, we present an approach to determine the minimal complexity of a control structure that is able to produce a given set of desired behaviors with a given morphology in a given environment. In other words, rather than comparing the complexities of the control structure and the behavior, we ask: what is the minimal brain complexity (or size) that can control all (desired) behaviors that are possible with the body in a given environment?

We follow a bottom-up–understanding by building–approach to cognitive science [17], which is also known as behavior-based robotics [18] and embodied artificial intelligence [1, 2]. The core concept is that cognitive systems are considered as embedded and situated agents which cannot be understood if they are detached from the sensorimotor loop. This implicitly means that we assume sensor state sparsity and continuity of physical constraints. Consider the human retina as an example. We do not see random images but structured patterns and, moreover, the sequence of these patterns is also highly dependent on our behavior. This behavior-dependent structuring of information is also known as *information self-structuring* and has been identified as one of the key principles of learning and development [19, 20]. The second implication from the sensorimotor loop is continuity, e.g. natural systems are unable to teleport themselves from one place to another. Therefore, we can safely assume that the world around us will not be too different from the recent past and the recent future.

**Fig 1:**
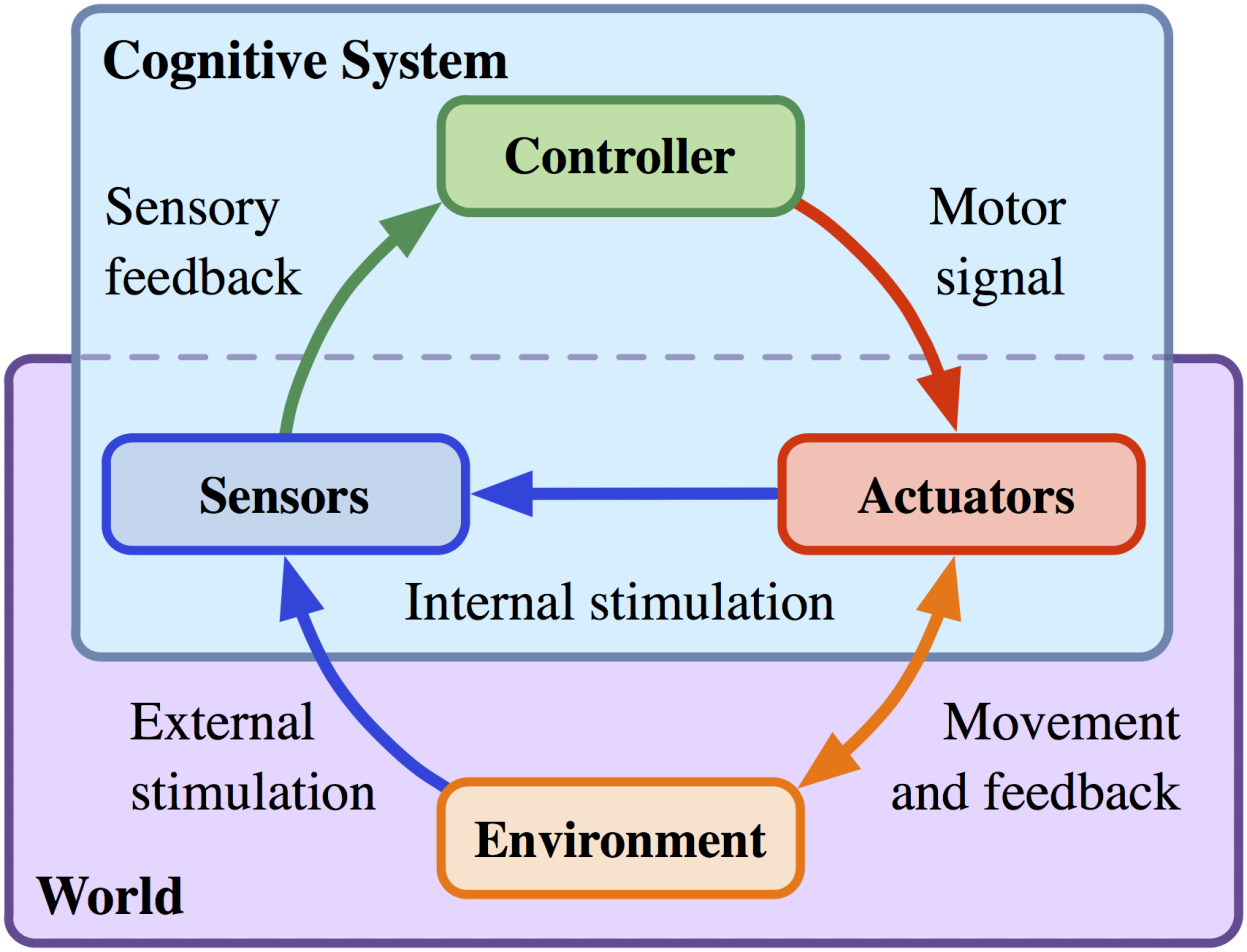
Sensorimotor loop.

The sensorimotor loop (SML) [21, 22] is described by a type of partially observable Markov decision process (POMDP) where an embodied agent chooses actions based on noisy partial observations of its environment. An illustration of this causal structure is given in Fig 1. We aim at optimizing the design of policy models for controlling these processes. One aspect of the optimal design problem is addressed by working out the optimal complexity of the policy model. In particular, we are interested in the minimal number of units or parameters needed in order to obtain an artificial neural network that can represent or approximate a desired set of behaviors. A first step towards resolving this problem is to address the minimal size of a universal approximator of policies. In realistic scenarios, universal approximation is out of question, since it demands an enormous number of parameters, many more than actually needed. In this paper we reconsider the universal approximation problem by exploiting embodiment constraints and restrictions in the desired behavioral patterns.

We introduce the notions of *embodied behavior dimension* and *embodied universal approximation*, which quantify the effective dimension of a system that is subject to sensorimotor constraints (embodiment) and formalize the minimal control paradigm of cheap design in the context of the sensorimotor loop. We substantiate these ideas with theoretical results on the representational capabilities of conditional restricted Boltzmann machines (CRBMs) as policy models for embodied systems. CRBMs are artificial stochastic neural networks where the input and output units are connected in a bipartite and undirected manner to a set of hidden units. Given the embodied behavior dimension, we derive bounds on the number of hidden units of CRBMs that is sufficient to generate a set of behaviors by appropriate tuning of interaction weights and biases. In order to test our theory, we present an experimental study with a six-legged walking robot and find a clear corroboration. The experiments indicate that the sufficient controller complexity bounds predicted by our theory are tight, which means that the theory has direct practical implications.

CRBMs are defined by clamping an *input* subset of the visible units of a restricted Boltzmann machine (RBM) [23, 24]. Conditional models of this kind have found a wide range of applications, e.g., in classification, collaborative filtering, and motion modeling [25–28], and have proven useful as policy models in reinforcement learning settings [29]. These networks can be trained efficiently [30, 31] and are well known in the context of learning representations and deep learning [32]. Although estimating the probability distributions represented by RBMs is hard [33], approximate samples can be generated easily from a finite Gibbs sampling procedure. The theory and in particular the expressive power of RBM probability models has been studied in numerous papers [34–37]. Recently also the representational power of CRBMs has been studied in detail [38]. CRBMs can model non-trivial conditional distributions on high-dimensional input-output spaces using relatively few parameters, and their complexity can be adjusted by simply increasing or decreasing the number of hidden units. Hence we chose this model class for illustrating our discussion about the complexity of SML control problems.

This paper is organized as follows. Section “The Causal Structure of the Sensorimotor Loop” contains a description of the sensorimotor loop. Section “From Policies to Behaviors” presents the notions of embodied behavior dimension and embodied universal approximation. In section “Cheap Representation of Embodied Behaviors” we use these two notions in order quantify and enforce dimensionality reduction. Section “A Case Study with Conditional Restricted Boltzmann Machines” contains our theoretical discussion on the representational power of CRBM models, comparing the non-embodied and the embodied settings. Section “Experiments with a Hexapod” validates the theory experimentally. In “Discussion” we offer our conclusions and outlook. In the Supporting Information S1–S4 Videos show the walking hexapod controlled by four CRBMs of different complexity, S1 Text contains technical proofs, S2 Text contains details about the estimation of the embodied behavior dimension, and S3 Text discusses possible generalizations of the ideas presented in the main part of the paper.

## Methods

### The Causal Structure of the Sensorimotor Loop

What is an embodied agent? In order to develop a theory of embodied agents that allows us to cast the core principles of the field of embodied intelligence into rigorous theoretical and quantitative statements, we need an appropriate formal model. Such a model should be general enough to be applicable to all kinds of embodied agents, including natural as well as artificial ones, and specific enough to capture the essential aspects of embodiment. How should such a model look like? First of all, obviously, an embodied agent has a body. This body is situated in an environment with which the agent can interact, thereby generating some behavior. In order to be useful, this behavior has to be guided or controlled by the agent’s brain or controller. Drawing the boundary between the brain on one side and the body, together with the environment, on the other side suggests a black box perspective of the brain. The brain receives sensor signals from and sends effector or actuator signals to the outside world. All it knows from the world is based on this closed loop of signal transmission. In other words, the world is a black box for the brain with which it interacts through sensing and acting. In particular, the boundary between the body and the environment is not directly “visible” for the brain. Both are parts of that black box and interact with the brain in an entangled way. Therefore, we consider them as being one entity, the outside world or simply the world. The brain is causally independent of the world, given the sensor signals, and the world is causally independent of the brain, given the actuator signals. This is the black box perspective.

Let us now develop a formal description of this sensorimotor loop. We denote the set of world states by 𝒲. This set can be, for instance, the position of a robot in a static 3D environment. Information from the world is transmitted to the brain through sensors. Denoting the set of sensor states by 𝒮, we can consider the sensor to be an information transmission channel from 𝒲 to 𝒮 as it is defined within information theory [39]. Given a world state *w* ∈ 𝒲, the response of the sensor can be characterized by a probability distribution *β*(*w*; *ds*) of possible sensor states *s* ∈ 𝒮 as result of *w*. For instance, if the sensor is noisy, then its response will not be uniquely determined. If the sensor is noiseless, that is, deterministic, then there will be only one sensor state as possible response to the world state *w*. In any case, the response of the sensor given *w* can be described in terms of a Markov kernel

$$\beta \;:\;𝒲\;⟶\;\Delta_{𝒮}\;,$$

where Δ_𝒮_ denotes the set of probability distributions on the set 𝒮 of sensor states. The set of all such sensor channels is denoted by $\Delta_{𝒮}^{𝒲}$. Whenever the base set 𝒮 is discrete, we simply write *s* instead of *ds* and *β*(*w*; *s*) instead of *β*(*w*; *ds*). Note that, as a Markov kernel, *β* satisfies various measure-theoretic conditions (positivity, normalization, measurability; for the technical definitions of Markov kernels see, e.g., [40]). Markov kernels are closely related to conditional probabilities, which would justify the notation *p*(*s*∣*w*) instead of *β*(*w*; *s*). However, we prefer the latter notation. Following Pearl’s concept of causal networks [41], the Markov kernels formalize the mechanisms of the sensorimotor loop. This means that the Markov kernels play the more fundamental role, and we want to distinguish this role also by the notation. Once the mechanisms are defined, they generate distributions so that we can also compute the conditional distributions from them. For instance, one could compute the conditional distribution *p*(*s*∣*w*) and compare this with the mechanism *β*(*w*; *s*). Clearly, they coincide whenever *p*(*w*) > 0, which we consider as a consistency property. However, there is an important difference, reflecting the fact that *β* is a mechanism: it is defined for *all* *w*. If the behavior of an agent is restricted to only a few world states, then the conditional distribution *p*(*s*∣*w*) will be defined only for these world states.

After having described the mathematical model of a sensor in detail, it is now straightforward to consider a corresponding formalization of the other components of the sensorimotor loop. We continue with the notion of a policy. The agent can generate an effect in the world in terms of its actuators. Since we consider the body as part of the world, this can lead, for instance, to some body movement of the agent. In order to guide this movement, it is beneficial for the agent to choose its actuator state based on the information about the world received through its sensors. Denoting the state set of the actuators by 𝒜, we can again consider a channel from 𝒮 to 𝒜 as formal model of a policy, represented by a Markov kernel

$$\pi \;:\;𝒮\;⟶\;\Delta_{𝒜}\;,$$

where Δ_*𝒜*_ denotes the set of probability distributions on 𝒜. Note that this definition of a policy also allows us to consider a random choice of actions, leading to so-called non-deterministic policies. The set of policies is denoted by $\Delta_{𝒜}^{𝒮}$. Finally, we consider the change of the world state from *w* to *w*′ in the context of an actuator state 𝒶 as a channel, denoted by *α*, which assigns a distribution *α*(*w*, *a*; *dw*′) to *w*, *a*. With the set Δ_𝒲_ of probability distributions on 𝒲, we have

$$\alpha \;:\;𝒲\times 𝒜\;⟶\;\Delta_{𝒲}\;.$$

We refer to *α* as *world channel* and denote the set of all world channels by $\Delta_{𝒲}^{𝒲\times 𝒜}$. Similar causal structures, involving Markov kernels, have been considered in the general context of control and systems theory [42] as well as in the context of population dynamics [43].

### From Policies to Behaviors

We have defined three mechanisms that are involved in a (reactive) sensorimotor loop of an embodied agent. Clearly, the agent’s embodiment poses constraints to this loop, which we attribute to the mechanisms *β* and *α*. The agent is equipped with these mechanisms, but they are both considered to be determined and not modifiable by the agent. On the other hand, the policy *π* can be modified by the agent in terms of learning processes. In order to describe the process of interaction of the agent with the world, we have to sequentially apply the individual mechanisms in the right order. Starting with an initial world state *w* ^*t*^ at time *t*, first the sensor state *s* ^*t*^ is generated in terms of the channel *β*. Then, based on the state of the sensor, an actuator state *a* ^*t*^ is chosen according to the policy *π*. Finally, the world makes a transition, governed by *α*, from the state *w* ^*t*^ to a new state *w* ^*t*+1^, which is influenced by the actuator state *a* ^*t*^ of the agent. Altogether, this defines the combined mechanism

$${\mathbb{P}}^{\pi }\left(w^{t};ds^{t},da^{t},dw^{t+1}\right)\;≔\;\beta \left(w^{t};ds^{t}\right)\;\pi \left(s^{t};da^{t}\right)\;\alpha \left(w^{t},a^{t};dw^{t+1}\right)\;.$$ (1)

**Fig 2:**
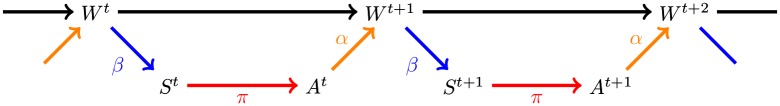
Causal structure of the reactive SML.

Note that we consider *β* and *α* fixed and therefore emphasize only the dependence on *π*. Now, with the new state *w* ^*t*+1^ of the world, the three steps are iterated. This generates a process which is shown in Fig 2. Formally, the process is a probability distribution over trajectories that start with *w* ^0^:

$$w^{0},\;s^{0},a^{0},w^{1},\;s^{1},a^{1},w^{2},\;s^{2},a^{2},w^{3},\;\ldots ,\;s^{T-1},a^{T-1},w^{T}\;.$$ (2)

In order to describe this probability distribution, we have to iterate the mechanism Eq (1) by multiplication:

$${\mathbb{P}}^{\pi }\left(w^{0};ds^{0},da^{0},dw^{1},\ldots ,ds^{T-1},da^{T-1},dw^{T}\right)\;≔\;\prod_{t=0}^{T-1}{\mathbb{P}}^{\pi }\left(w^{t};ds^{t},da^{t},dw^{t+1}\right)\;.$$ (3)

Now, what aspects of the sequence Eq (2) represent the behavior of the agent? Let us consider, for instance, a walking behavior. It is given as a movement of the agent’s body in physical space, which is completely determined by the world process. Remember that the body is part of the world. Clearly, the particular sequence of sensor and actuator states does not matter as long as they contribute to the generation of the same body movement. Therefore, we consider the world process *w* ^*t*^ as the one in which behavior takes place and marginalize out the other processes from Eq (3), which leads to:

$${\mathbb{P}}^{\pi }\left(w^{0};dw^{1},\ldots ,dw^{T}\right)\;.$$ (4)

One can show that, with weak assumptions, the limit for *T* → ∞ exists, so that we can write

$${\mathbb{P}}^{\pi }\left(w^{0};dw^{1},dw^{2},\ldots \right)\;,$$

which is a Markov kernel from an initial world state *w* ^0^ to the set of all infinite future sequences *w* ^1^, *w* ^2^, …. Denoting the set of such Markov kernels by $\Delta_{{𝒲}^{\infty }}^{𝒲}$, we can formalize the *policy-behavior map*, which assigns to each policy the corresponding behavior:

$$\psi_{\infty }\;:\Delta_{𝒜}^{𝒮}\;\;⟶\;\;\Delta_{{𝒲}^{\infty }}^{𝒲},\;\pi \;\;\mapsto \;\;{\mathbb{P}}^{\pi }\left(w^{0};dw^{1},dw^{2},\ldots \right)\;.$$ (5)

Two policies *π*_1_ and *π*_2_ will be considered equivalent, if they generate the same behavior, that is,

$$\psi_{\infty }\left(\pi_{1}\right)\;=\;\psi_{\infty }\left(\pi_{2}\right)\;.$$ (6)

We argue that embodiment constraints render many policies equivalent. We can exploit this fact in order to design a concise control architecture. This will lead to a quantitative treatment of the notion of cheap design within the field of embodied intelligence. Let us treat this systems design problem in a more rigorous way. As we pointed out, the agent is equipped with the mechanisms *β* and *α* which constitute the embodiment of the agent. In a biological system these mechanisms will change due to developmental processes. However, we want to restrict our attention to the learning processes and disentangle them from developmental processes by assuming that the latter ones have already converged and therefore consider them as fixed. Learning refers to a process in which the policy is changing in time. Clearly, in order to model this change the agent has to be equipped with a family of possible policies, which we denote by 𝓜, and refer to as *policy model*. For instance, we can consider a neural network as a policy model that is parametrized by synaptic weights and threshold values for the individual neurons. Changing the weights and the thresholds will lead to a change of the policy (although there may be degeneracies, in general). In any case, going through all the possible parameter values will generate a set 𝓜 of policies with which the agent is equipped for its behavior.

Intuitively, it is clear that the embodiment constraints cause restrictions in the set of behaviors that an agent can realize. For example, inertia restricts the pace at which an embodied system can change its direction of motion (imagine a train switching the traveling direction instantaneously). In turn, not all world-state transitions may be possible in a single time step, regardless of what the policy specifies as a desirable action to take. These restrictions create a bottleneck between the set of policies on one side and the set of possible behaviors on the other. The consequence is that, generically, infinitely many policies parametrize the same behavior, in the sense of Eq (6). Therefore, it should be possible to find a concise model 𝓜 that is capable of generating all possible behaviors. In particular, we consider a model 𝓜 that satisfies

$$\psi_{\infty }\left(\bar{𝓜}\right)\;=\;\psi_{\infty }\left(\Delta_{𝒜}^{𝒮}\right)\;,$$

where $\bar{𝓜}$ denotes the set of limit points of 𝓜. We refer to this property of 𝓜 as being an *embodied universal approximator*. In order to highlight the exploitation of embodiment constraints for cheap design, we compare this kind of universal approximation to the standard notion of universal approximation, $\bar{𝓜}=\Delta_{𝒜}^{𝒮}$, which we refer to as *non-embodied universal approximation*.

### Cheap Representation of Embodied Behaviors

If we understand the way in which different policies are mapped to the same, or to different, behaviors, then we can parametrize all the behaviors that can possibly emerge in the SML by a low-dimensional (or low-complexity) set of policies. We develop the necessary tools in this section. For clarity we will focus on the reactive SML with *finite* sensor and actuator state spaces but allowing the possibility of a continuous world state. In particular we will use *β*(*w*; *s*) instead of *β*(*w*; *ds*) and *π*(*s*; *a*) instead of *π*(*s*; *da*). Possible generalizations of these settings are discussed in supporting information S3 Text. See S3 Fig for an illustration of a generalization to non-reactive systems.

For a reactive SML, the condition stated in Eq (6) is equivalent to

$${\mathbb{P}}^{\pi_{1}}\left(w;dw^{\prime }\right)\;=\;{\mathbb{P}}^{\pi_{2}}\left(w;dw^{\prime }\right)\;,$$

where

$${\mathbb{P}}^{\pi }\left(w;dw^{\prime }\right)=\sum_{s\in 𝒮}\sum_{a\in 𝒜}\beta \left(w;s\right)\pi \left(s;a\right)\alpha \left(w,a;dw^{\prime }\right)$$ (7)

is the one-step world state transition kernel. Therefore, the mechanism ℙ^*π*^(*w*; *dw*′) will play an important role in our analysis and we consider the one-step formulation of the policy-behavior map:

$$\psi :\Delta_{𝒜}^{𝒮}\;\;⟶\;\;\Delta_{𝒲}^{𝒲},\;\pi \;\;\mapsto \;\;{\mathbb{P}}^{\pi }\left(w;dw^{\prime }\right)\;.$$ (8)

The map *ψ* is an affine map from the convex set $\Delta_{𝒜}^{𝒮}$ to the convex set $\Delta_{𝒲}^{𝒲}$. Its image

$$𝔅≔\psi (\Delta_{𝒜}^{𝒮})$$

represents the set of all possible behaviors that the SML can generate. We refer to the dimension of 𝔅 as the *embodied behavior dimension*

d≔dim(𝔅).

The embodied behavior dimension *d* is equal to the maximal number of affinely independent vectors in the set 𝔅. This is given by the number of linearly independent vectors in the set

$$\beta \left(w;s\right)(\alpha \left(w,a_{0};dw^{\prime }\right)-\alpha \left(w,a;dw^{\prime }\right)),\;s\in 𝒮\text{,}\;a\in 𝒜\setminus \{a_{0}\}\text{,}$$ (9)

for some arbitrary *a*_0_ ∈ 𝒜. See supporting information S1 Text for more details about this.

In order to illustrate the effect of the embodiment on the embodied behavior dimension *d*, we formulate a simple upper bound in terms of *β* and *α*. In order to do so, we interpret the expression Eq (9) as a product of two vectors. More precisely, for each *s* ∈ 𝒮 consider the vector *β*(⋅; *s*) that assigns to each *w* ∈ 𝒲 the number *β*(*w*; *s*), and denote by rank(*β*) the maximal number of linearly independent vectors *β*(⋅; *s*). If 𝒲 is finite, rank(*β*) is simply the rank of the matrix with entries (*β*(*w*; *s*))_*w* ∈ 𝒲, *s* ∈ 𝒮_. Furthermore, for each *a* ∈ 𝒜 ∖ {*a*_0_} consider the difference vector *α*(⋅, *a*_0_; *dw*′) − *α*(⋅, *a*; *dw*′) that assigns to each *w* the difference *α*(*w*, *a*_0_; *dw*′) − *α*(*w*, *a*; *dw*′) of probability distributions. Let rank(*α*) denote the maximal number of linearly independent difference vectors of that form. If 𝒲 is finite, rank(*α*) is simply the rank of the matrix with entries (*α*(*w*, *a*_0_; *w*′) − *α*(*w*, *a*; *w*′))_*a* ∈ 𝒜∖{*a*_0_},(*w*, *w*′) ∈ 𝒲 ×𝒲_.

With these definitions, Eq (9) yields

d≤rank(β)·rank(α). (10)

The upper bound Eq (10) may not provide an accurate estimate of the embodied behavior dimension. However, it illustrates how the embodiment constraints, represented by *β* and *α*, can lead to an embodied behavior dimension *d* that is much smaller than the dimension of the set of all policies $\Delta_{𝒜}^{𝒮}$, which is ∣𝒮∣(∣𝒜∣ − 1). In the following, we present simple examples that illustrate why the rank of *β* and *α* is expected to be small in embodied systems.

**Fig 3:**
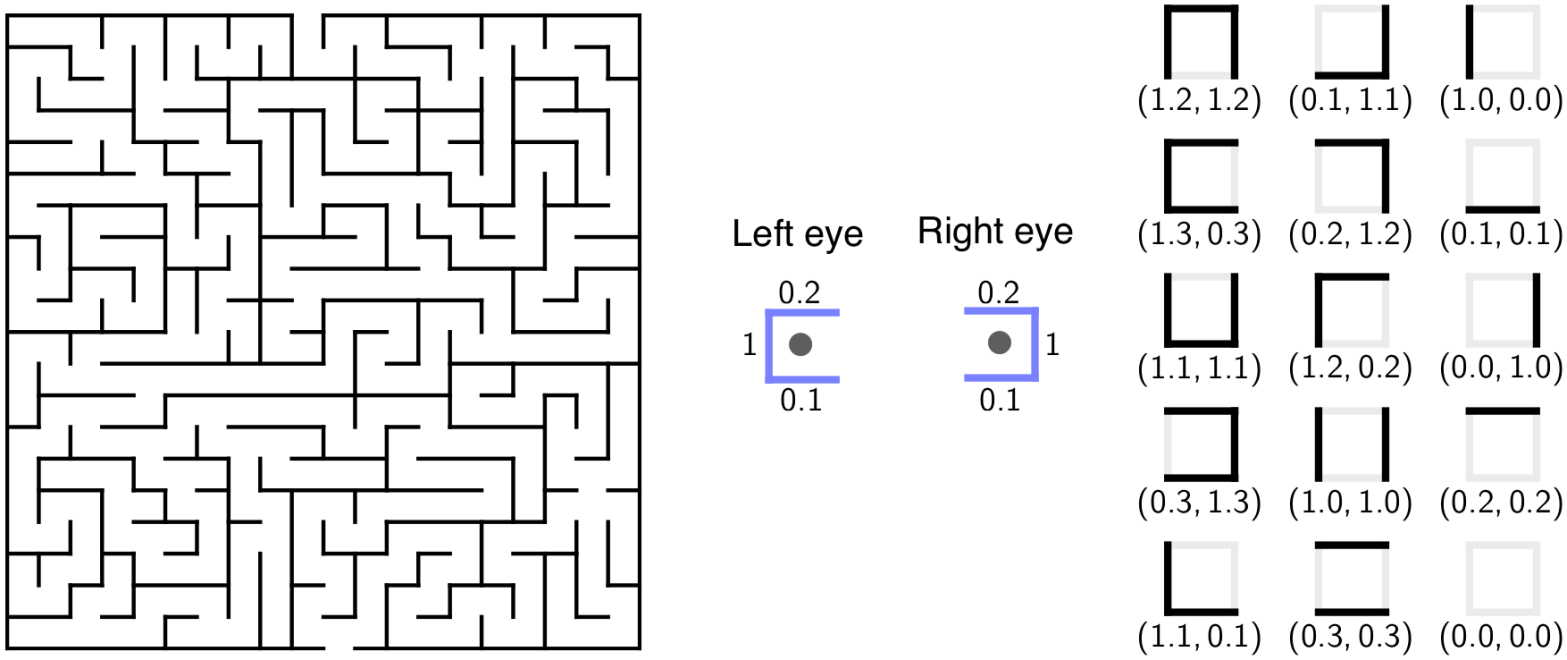
Ambiguity and redundancy of the sensor measurement. In this example, an agent navigates the 20 × 20 maze shown in the left panel. The agent is endowed with two sensors (eyes), *S*_left_ and *S*_right_. Each sensor measures a weighted average of the walls in the immediate vicinity, illustrated in the central panel, and outputs one of 8 possible numerical values, as shown in the right panel. There are 400 possible locations in the maze but only 8 × 8 = 64 joint sensor states. This implies that the sensor measurement is highly ambiguous about the world state. Furthermore, the outputs of both sensors are not independent; they always have the same value at the decimal place. Due to this redundancy, the factual number of joint sensor states is 15, instead of 64.

The sensors are usually insensitive to a large number of variations of the world state *w*. This means that *β* outputs the same distribution for several different *w*. Furthermore, the sensors implement a certain degree of redundancy, meaning that, for each 𝒲 the probability distribution *β*(*w*; ⋅) ∈ Δ_𝒮_ has certain types of symmetries. Consider, for example, the 20 × 20 maze shown in Fig 3 (left-hand side). The world state includes the location of the agent in the maze, (*i*, *j*) ∈ {1, …, 20}^2^. The agent is endowed with two sensors: a left eye and a right eye. Each eye measures a weighted sum of the light intensity arriving from the walls in the immediate vicinity of the agent. The left eye outputs the value *S*_left_ = 0.8 *x*_*w*_ + 0.2 *x*_*n*_ + 0.1 *x*_*s*_ + 0 *x*_*e*_ (with probability one), where *x*_*w*,*n*,*s*,*e*_ = 1 if there is a wall to the immediate west, north, south, east, respectively, and 0 otherwise. Similarly, *S*_right_ = 0 *x*_*w*_ + 0.2 *x*_*n*_ + 0.1 *x*_*s*_ + 0.8 *x*_*e*_. Each eye can produce a total of 8 states: 0,0.1,0.2,0.3,1,1.1,1.2,1.3. The naive number of joint sensor states (*S*_left_, *S*_right_) is 8 × 8 = 64. However, both eyes are partially redundant, and the actual total number of possible joint states is 15 (the case of four walls surrounding a location is excluded). In this example, 400 world states are mapped onto 15 sensor states, which implies that the rank of *β* is 15. This example illustrates the two typical properties of the sensor measurement mentioned above: the ambiguity of the measurement, mapping several world states to the same sensor state, and the redundancy, by which several sensors measure partially overlapping information about the world state.

**Fig 4:**
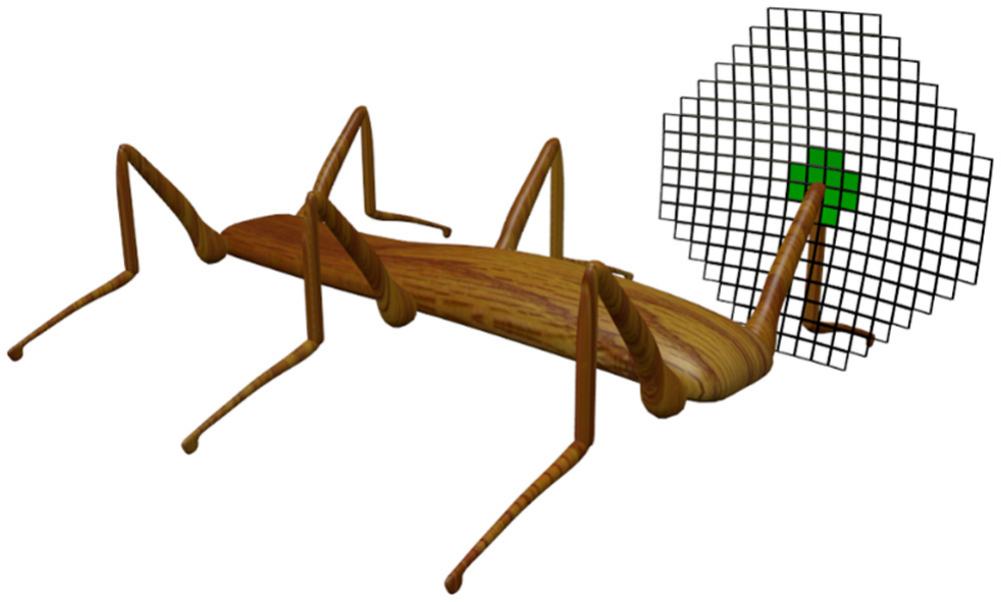
Locality of world-state transitions. At subsequent time steps, the knee of a robot can only move by a small amount. Only very few world state transitions are possible within one time step (e.g., transitions to neighboring positions). This hexapod is used in the experimental evaluation of our theory in “Experiments with a Hexapod”.

In the case of *α*, usually several actions *a* produce the same world state transition, such that, for any fixed world state *w*, *α*(*w*, ⋅; ⋅) is piece-wise constant with respect to *a*. Furthermore, for any given *w*, only very few states *w*′ ∈ 𝒲 are possible at the next time step, regardless of *a*, such that *α*(*w*, *a*; ⋅) assigns positive probability only to a very small subset of 𝒲. This means that rank(*α*) is usually much smaller than (∣𝒜∣ − 1) (the maximum theoretically possible rank). An example for this kind of constraints on *α* is a robot’s knee, which in a time step can only be moved to adjacent positions, as the one shown in Fig 4.

So far we have discussed the embodied behavior dimension of an embodied system and reasoned why it can be much smaller than the dimension of the policy space. Since the policy-behavior map *ψ* is affine, for any generic behavior that can possibly emerge in the SML, there is a $\tilde{d}$-dimensional set (in fact a polytope) of equivalent policies generating that same behavior, where $d+\tilde{d}$ equals the full dimension ∣𝒮∣(∣𝒜∣ − 1). By selecting representatives from each set of equivalent policies, we can define low-dimensional policy models which are just as expressive as the much higher dimensional set $\Delta_{𝒜}^{𝒮}$ of all possible policies, in terms of the representable behaviors. The following example shows that it is possible to define a smooth manifold of policies which translate in a one-to-one fashion to the set of all possible behaviors in the SML.

**Fig 5:**
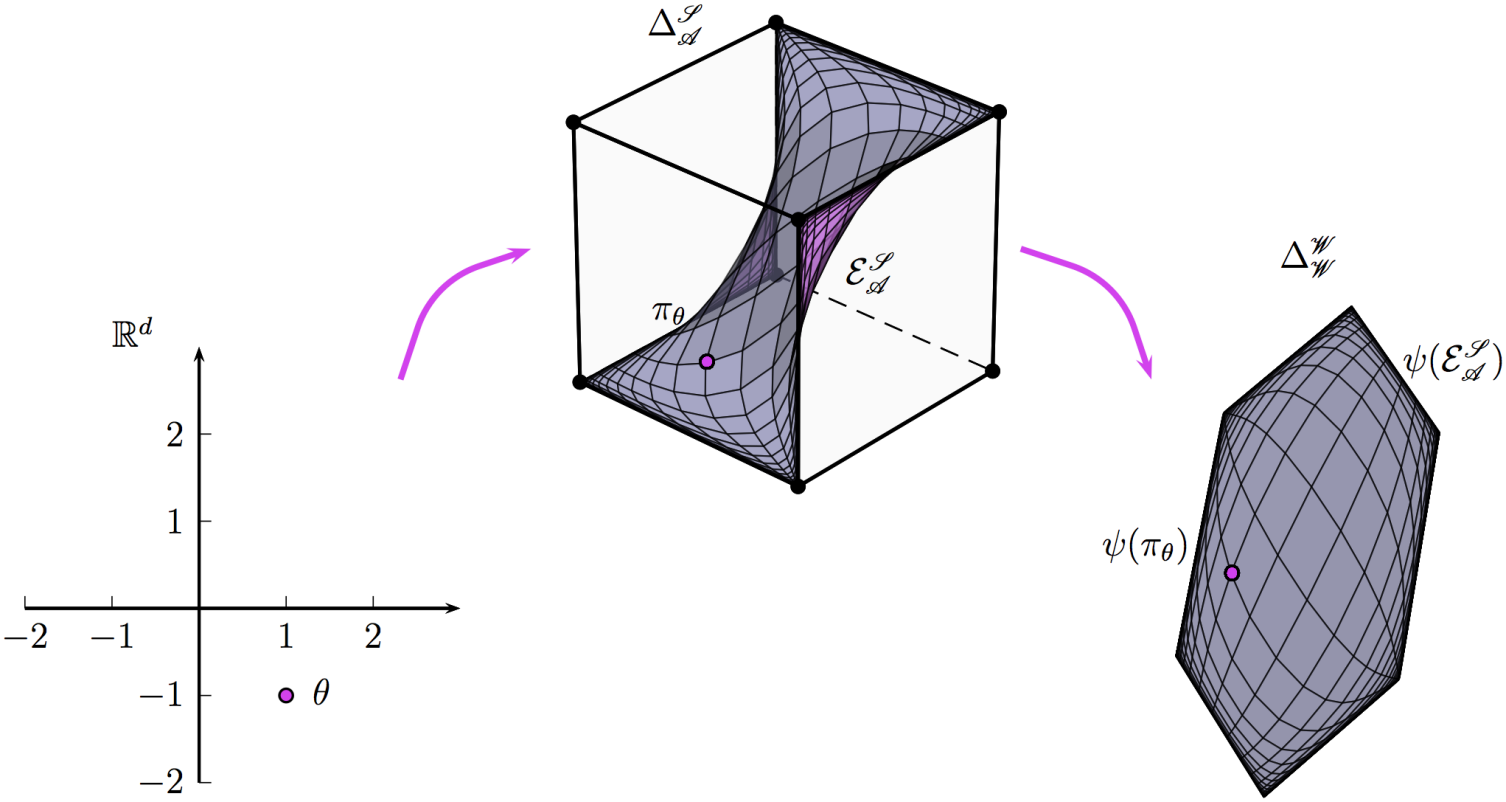
Illustration of the exponential family Eq (11) of policies. This figure shows an example with ∣𝒲∣ = 3 and ∣𝒜∣ = 2 and a policy-behavior map *ψ* with embodied behavior dimension *d* = 2. In this case, the polytope $\Delta_{𝒜}^{𝒮}$ is the three-dimensional cube of 3 × 2 row stochastic matrices shown in the middle. The curved surface within is the exponential family ${𝓔}_{𝒜}^{𝒮}$, which is parametrized by two parameters. The exponential family is mapped by the policy behavior map *ψ* to the same set of behaviors (the hexagon illustrated in the right) as the set $\Delta_{𝒜}^{𝒮}$ of all policies.

**Example. Embodied universal approximator of minimal dimension.** Consider the matrix *E* ∈ ℝ^*d*×(𝒮×𝒜)^ that represents the policy-behavior map *ψ* with respect to some basis. Then the exponential family ${𝓔}_{𝒜}^{𝒮}$ of policies defined by

$$\pi_{\theta }\left(s;a\right)=\frac{\exp(\theta^{⊤}E\left(s,a\right))}{\sum_{a^{\prime }\in 𝒜}\exp\left(\theta^{⊤}E\left(s,a^{\prime }\right)\right)},\;\theta \in {\mathbb{R}}^{d}\;,$$ (11)

is an embodied universal approximator of dimension *d*. In fact, each behavior from the set 𝔅 is realized by exactly one limit point of the set ${𝓔}_{𝒜}^{𝒮}$. See Fig 5 for an illustration and supporting information S1 Text for technical details.

The previous discussion shows that the set of behaviors 𝔅 that can possibly emerge in the SML usually has a much lower dimension than the set of all policies. Furthermore, it shows that it is possible to construct low-dimensional embodied universal approximators. Nonetheless, among all behaviors that are possible in the SML, we can expect that only a smaller subset 𝓑 ⊆ 𝔅 is actually relevant to the agent. For instance, among all locomotion gaits that an agent could possibly realize with its body in a given environment, we can expect that it will only utilize those which are most successful (e.g., in terms of maximizing some reward function or the predictive information [44–46]). The notion of embodied behavior dimension can be directly generalized in order to accurately account for such behavioral restrictions. Given any set of behaviors 𝓑, we are interested in the following problem.

**Problem.** For a given set of behaviors $𝓑\subseteq 𝔅=\psi (\Delta_{𝒜}^{𝒮})$ and a class 𝔐 of policy models, what is the smallest model 𝓜 ∈ 𝔐 that can generate all these behaviors; that is, $𝓑\subseteq \psi (\bar{𝓜})$?

Later below we will consider a class 𝔐 of policy models defined in terms of CRBMs. In what follows, we focus on sets 𝓑 of behaviors that take place within a subset 𝓦 ⊆ 𝒲 of world states and consider the corresponding subset 𝓢≔{s∈𝒮:s∈supp(β(w;⋅))forsomew∈𝓦} of sensor values that can be observed at these world states. When controlling behaviors from the set 𝓑, only states in 𝓢 are relevant for the policy, as the other sensor states are never observed. Furthermore, in order to stay in 𝓦 only a restricted set 𝓐_*s*_ of actuator states is allowed given that the state 𝒲 is observed. This motivates us to study a set of policies that is assigned to a sensor state set and a family of corresponding positive probability actuator state sets. In order to simplify the notation, we denote the family 𝓐_*s*_, *s* ∈ 𝓢, simply by 𝓐 and consider the set $\Delta_{𝓐}^{𝓢}\subseteq \Delta_{𝒜}^{𝒮}$ of policies *π* that satisfy

$$s\in 𝓢,\;\pi \left(s;a\right)>0\;\to \;a\in {𝓐}_{s}\;.$$

Next we consider the set ${𝓑}^{𝓢,𝓐}≔\psi (\Delta_{𝓐}^{𝓢}){|}_{𝓦}$ of behaviors on 𝓦 that are generated by policies with the given sensor and actuator restrictions. With its dimension *d* ^𝓢,𝓐^: = dim(𝓑^𝓢,𝓐^), we have the following result, which gives us a simple and powerful combinatorial tool for addressing the above problem of representing specific behavior sets.

**Lemma 1.** *Any model* $𝓜\subseteq \Delta_{𝒜}^{𝒮}$ *with the following property can approximate any behavior from the set* 𝓑^𝓢,𝓐^ *arbitrarily well: for every policy* $\pi \in \Delta_{𝒜}^{𝒮}$ *whose* 𝓢-*rows have a total of* ∣𝓢∣ + *d* ^𝓢,𝓐^ *or less non-zero entries, there exists a policy* $\pi^{*}\in \bar{𝓜}$ *with* *π*(*s*; ⋅) = π*(*s*; ⋅) *for all* *s* ∈ 𝓢.

See supporting information S1 Text for technical details and S1 Fig for an illustration of this result. This lemma states that for universal approximation of embodied behaviors it suffices to approximate the policies that assign positive probability only to a limited number of actions (for a relevant set of sensor values). The number of actions is determined by the embodied behavior dimension. Keep in mind that the relevant set 𝓢 of sensor values may be much smaller than 𝒲 not only due to behavioral constraints but also due to the redundancy of the sensors. Recall the maze example from Fig 3, where there are 64 theoretically possible sensor values but only 15 that are actually measured. This kind of redundancy is typical for embodied systems and generally leads to a strong reduction of the sensor states. Furthermore, redundancy in the sensor process results not only from the nature of the sensory apparatus, as in the maze example, but also from the agent’s behavior. This important mechanism, which is exploited by embodied agents, is known as information self-structuring [20].

It is worthwhile mentioning that the exponential family from Eq (11) and Lemma 1 describe two complementary types of universal approximators of embodied behaviors. The first type, described in the example, is composed of maximum entropy policies, whereas the second type, described in the lemma, is composed of minimum entropy policies. If we consider the set of equivalent policies that map to a given behavior, the exponential family selects the one with the most random state-action assignments that are possible for generating that behavior. On the other hand, Lemma 1 selects the ones with the most deterministic state-action assignments that are possible for generating that behavior. Geometrically, the set of equivalent policies of a given behavior is the convex hull of the minimum entropy policies, with the maximum entropy policy lying in the center. The exponential family has nice geometric properties, but it is very specific to the kernels *β* and *α*, which define the sufficient statistics *E*. The set described in Lemma 1 can also be considered as a policy model. It offers several advantages that we will exploit later on. First, it has a very simple combinatorial description. Second, it only depends on the embodied behavior dimension *d*, irrespective of the specific kernels *β* and *α* (which are not directly accessible to the agent). Third, it selects policies with the minimum possible number of positive probability actions.

## Results

### A Case Study with Conditional Restricted Boltzmann Machines

#### Definitions

A Boltzmann machine (BM) is an undirected network of stochastic binary units, some of which may be hidden. Such a network defines probabilities for the joint states of the visible units, given by the relative frequencies at which these states are observed, asymptotically, depending on the network parameters (interaction weights and biases). The probability of each joint state *x* = (*x*_*V*_, *x*_*H*_) of the visible and hidden units is given by the Gibbs-Boltzmann distribution $p(x)=\frac{1}{Z}\text{exp}(-𝓗(x))$ with energy function 𝓗(*x*) = ∑_*i*,*j*_ *x*_*i*_ *W*_*ij*_ *x*_*j*_ + ∑_*i*_ *b*_*i*_ *x*_*i*_ and normalization partition function *Z*(*W*, *b*) = ∑_*x*′_ exp(−𝓗(*x*′)). The probabilities of the visible states are given by marginalizing out the states of the hidden units, *p*(*x*_*V*_) = ∑_*x*_*H*__ *p*(*x*_*V*_, *x*_*H*_).

An RBM is a BM with the restriction that there are interactions only between the visible and the hidden units; that is, *W*_*ij*_ ≠ 0 only when unit *i* is visible and *j* hidden. An RBM defines a model of conditional probability distributions, given by clamping the states of some of the visible units:

**Definition 2.** The conditional restricted Boltzmann machine model with *k* input, *n* output, and *m* hidden units, denoted ${\text{CRBM}}_{n,m}^{k}$, is the set of all conditional distributions in $\Delta_{𝒜}^{𝒮}$, 𝒮 = {0,1}^*k*^, 𝒜 = {0,1}^*n*^, that can be written as

$$\begin{array}{c} \pi \left(s;a\right)=\frac{1}{Z(W,b,Vs+c)}\sum_{z\in {\left\{0,1\right\}}^{m}}\exp\left(z^{⊤}Wa+z^{⊤}Vs+b^{⊤}a+c^{⊤}z\right),\;\forall a\in {\left\{0,1\right\}}^{n},s\in {\left\{0,1\right\}}^{k}\;, \end{array}$$

where ⊤ denotes vector transposition and *Z*(*W*, *b*, *Vs* + *c*) is a normalization factor for each *s* ∈ {0,1}^*k*^. Here, *s*, *a*, and *z* are state vectors of the input, output, and hidden units, respectively. Furthermore, *V* ∈ ℝ^*m*×*k*^ is a matrix of interaction weights between hidden and input units, *W* ∈ ℝ^*m*×*n*^ is a matrix of interaction weights between hidden and output units, *c* ∈ ℝ^*m*^ is a vector of biases for the hidden units, and *b* ∈ ℝ^*n*^ is a vector of biases for the output units.

**Fig 6:**
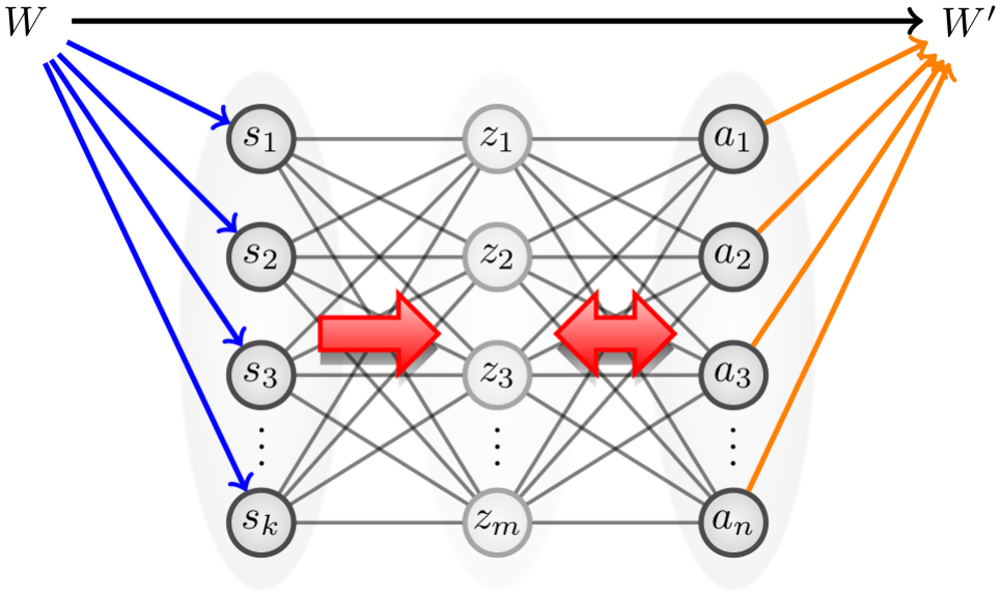
Illustration of a CRBM policy in the sensorimotor loop.

The model ${\text{CRBM}}_{n,m}^{k}$ has *mk* + *mn* + *m* + *n* parameters (the interaction weights and biases). When there are no input units, i.e., *k* = 0, the conditional probability model reduces to the restricted Boltzmann machine probability model with *n* visible and *m* hidden units, which we denote by RBM_*n*,*m*_. Fig 6 illustrates a CRBM in the sensorimotor loop.

#### Non-embodied vs. embodied universal approximation

In this section we present results about the representational power of CRBMs contrasting the minimal number of hidden units that suffices for non-embodied universal approximation with the minimal number of hidden units that suffices for universal approximation of embodied behaviors. In the first case we ask for the minimal *m* for which the model ${\text{CRBM}}_{n,m}^{k}$ can approximate every conditional distribution from the set $\Delta_{𝒜}^{𝒮}$ with 𝒮 = {0,1}^*k*^ and 𝒜 = {0,1}^*n*^, denoted by $\Delta_{n}^{k}$, arbitrarily well. We have the following result:

**Theorem 3.** **Non-embodied universal approximation.** *The model* ${\text{CRBM}}_{n,m}^{k}$ *can approximate every conditional distribution from* $\Delta_{n}^{k}$ *arbitrarily well if* $m\geq \frac{1}{2}2^{k}(2^{n}-1)$ *and only if* $m\geq \frac{1}{\left(n+k+1\right)}(2^{k}(2^{n}-1)-n)$.

The full statement of the theorem is quite technical, and thus we refer the interested reader to [38]. The necessary bound (the second inequality), however, follows from simple parameter counting arguments. The result shows that the number of hidden units required for non-embodied universal approximation is exponential in the number of input and output units.

Now we take a look at the embodied setting. By Lemma 1, we can achieve embodied universal approximation by considering only policies with a limited number of non-zero entries. Using each hidden unit of a CRBM to model each relevant non-zero entry of the policy, we obtain the following result:

**Theorem 4.** **Embodied universal approximation.** *The model* ${\text{CRBM}}_{n,m}^{k}$ *can approximate any behavior from* 𝓑^𝓢,𝓐^ *arbitrarily well whenever* *m* ≥ ∣𝓢∣ + *d* ^𝓢,𝓐^ − 1. *In particular, the model is an embodied universal approximator whenever* *m* ≥ ∣𝒮∣ + *d* − 1.

*Proof*. We use Lemma 1. The joint probability model RBM_*k*+*n*,*m*_ can approximate any probability distribution with support of cardinality *m* + 1 arbitrarily well [34, 35]. Hence, with *m* ≥ ∣𝒮∣ + *d* − 1, RBM_*k*+*n*,*m*_ can approximate any joint distribution with ∣𝒮∣ + *d* non-zero entries arbitrarily well. The result for conditional distributions is a direct implication of this.

This theorem gives an upper bound for the minimal number of hidden units that suffices to obtain embodied universal approximation. The bound depends on the embodiment constraints of the system, captured in the embodied behavior dimension. In general, this bound will be much smaller than the exponential bound from Theorem 3.

### Experiments with a Hexapod

**Fig 7:**
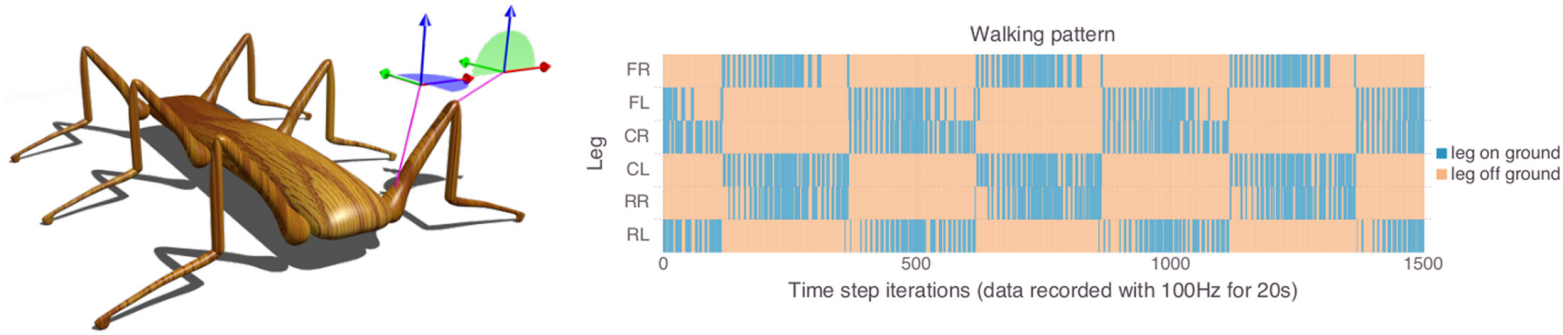
Hexapod set-up. Left-hand side: The simulated hexapod with a display of the joint configurations. Right-hand side: Visualization of the target walking pattern. The plot shows which leg touched the ground at which point in time. Blue areas refer to a contact with a the ground, while orange areas refer to points in time during which the correspond leg did not touch the ground. The different legs are plotted over the *y*-axis, while each point on the *x*-axis refers to a single point in time.

In the previous sections we have derived a theoretical bound for the complexity of a CRBM based policy. In this section, we want to evaluate that bound experimentally. For this purpose, we chose a six-legged walking machine (hexapod) as our experimental platform (see Fig 7 left panel), because it has a well-studied morphology in the context of artificial intelligence, with one of its first appearances as Ghengis [47]. The purpose of this section is *not* to develop an optimal walking strategy for this system. Contrary, this morphology was chosen, because the tripod gait (see Fig 7 right panel) is known to be one of the optimal locomotion behaviors, which can be implemented efficiently in various ways. This said, learning a control for this gait is not trivial, and hence it is a good testbed to evaluate our complexity bound for CRBM based policies.

This section is organized in three parts. The first part presents the experimental set-up as far as it is required to understand the results. The second part describes how the CRBM complexity parameter *m* was estimated form the data. The third part presents the results of the experiment and compares them with the theoretical bound.

#### Simulation

The hexapod was simulated with YARS [48], which is a mobile robot simulator based on the bullet physics engine [49]. Each segment of the hexapod is defined by its physical properties (dimension, weight, etc.). In the case of the hexapod shown in Fig 7 (left-hand side), the main body’s dimension (bounding box) is 4.4m length, 0.7m width, 0.5m height, and the weight is 2kg. For the tripod walking gait, inertia does not play a significant role, which means that the effect of the specific weight of the hexapod is negligible. Each leg consists of three segments (femur, tarsus, tibia). However, the two lower segments (tarus, tibia) are connected by a fixed joint. The leg segments were freely modeled with respect to the dimensions of an insect leg. The motors that connect a femur and tarsus (knee) only allow rotations around the local *y*-axis of the femur segment (see Fig 7). The deviation of the knee joints is limited to *ω*_knee_ ∈ [−15°,25°]. For joints which connect the main body with a femur (shoulder), the deviation is limited to *ω*_shoulder_ ∈ [−35°,35°]. The rotation axes of the shoulder joints are limited to the local *z*-axis of the main body. The angular positions of the joints define the sensor (${\tilde{S}}_{i}$) and actuator (${\tilde{A}}_{i}$) values. The actuator values (output of the CRBM) are translated into forces, which are controlled by bullet and YARS to reach the desired angular position (for details see [48, 49]). The maximal joint forces are large enough, such that each leg is able to lift the entire body on its own. This is done to mimic the leg strength of insects.

The policy update frequency was set to 10Hz, i.e., every 100ms the controller received a sensor value per sensor and generated an actuator value per actuator. The target behavior of the hexapod (a tripod walking gait, see Fig 7) was generated by an open-loop controller which applied phase shifted sine oscillations to the actuators. For each actuator, one period of the corresponding sine oscillation was discretized into 50 values (a single locomotion step requires 5 seconds). A faster walking behavior would not have given the legs enough time to reach the maximal angular positions. For evaluating the bound, exploiting the maximal range of the sensors and actuators is more important than optimizing the walking speed of the hexapod.

For the training and analysis, the sensor and actuator data was discretized into 16 uniform bins for each sensor and actuator. This corresponds to four binary input units for each sensor and four binary output units for each actuator. Combined into two random variables *S* = (*S*_1_, *S*_2_, …, *S*_12_), *A* = (*A*_1_, *A*_2_, …, *A*_12_), this leads to a total of 16^12^ possible values (∣𝒮∣ = ∣𝒜∣ = 16^12^) corresponding to a total of 48 binary input and 48 binary output units. In the following sections, we only refer to this preprocessed data, which means that calculations and the training of the CRBMs described in the remainder of this section refer to the two random variables *S*, *A*.

In the next section we estimate the controller complexity that is sufficient to reproduce the desired tripod walking gait.

#### Estimation of the sufficient complexity

Before the estimation procedure and results are presented, we restate the inequality given in Theorem 4, which is given by

$$m\geq \left|𝓢\right|+d^{𝓢,𝓐}-1\;.$$ (12)

This means that a CRBM should not require more hidden units (*m*) than the sum of the support set cardinality ∣𝓢∣ and embodied behavior dimension *d* ^𝓢,𝓐^ minus 1. The following paragraphs explain how these two values were calculated from the recorded data.

The first step in estimating the sufficient controller complexity of the CRBM policy model is the estimation of the support’s cardinality ∣𝓢∣. It was mentioned above that there are 16^12^ possible sensor values. The necessary complexity of a CRBM policy for a specific behavior depends on the actually used number of sensor values, which we call the sensor support set. By estimating the cardinality of the support set, we know how many sensor values the CRBM needs to take into account in order to reproduce the behavior of interest.

**Fig 8:**
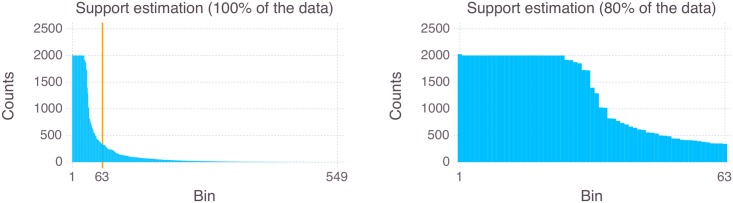
Estimation of the support’s cardinality. Estimation of the support set cardinality (before and after pruning).

The estimation of the support set cardinality depends on the quality of the sample. Therefore, we sampled the sensor and actuator values of the target behavior over 10^5^ time steps to ensure a sufficient convergence of the relative frequencies. Fig 8 (left-hand side) shows the histogram for all recorded sensor values. The orange vertical line shows where we have pruned the data so that 80% of the recorded data was kept. Fig 8 (right-hand side) shows the remaining data. With this procedure (recording, estimating relative frequencies, pruning the data to 80%), we estimated the cardinality of the sensor support set at ∣𝓢∣ = 63. The pruning threshold of 80% might appear arbitrary here. To clarify, estimating the support from data is an interesting research topic by itself, which, however, goes beyond the scope of this work. Our underlying assumption for the pruning is that the sampling is noisy. We decided for a pruning threshold at approximately twice the inflection point of the histogram. We want to point out that the threshold was chosen before the results of the experiments (see next section) were available.

The next step is to estimate the embodied behavior dimension, which is done here based on the affine rank of the empirically estimated internal world model *γ*(*s*, *a*; *s*′). For the sake of readability, we defer the justification for the replacement of the embodiment-behavior dimension by the affine rank of the internal world model to the supporting information S2 Text. See S2 Fig for an illustration of the underlying causal structure.

Given the internal world model $\tilde{\gamma }$ sampled from the target behavior, we obtain the relevant quantity *d* ^𝓢,𝓐^ as follows (see supporting information S2 Text):

$$d^{𝓢,𝓐}=\sum_{s\in 𝓢}\mathrm{rank}\left({\left(\tilde{\gamma }\left(s,a_{0};s^{\prime }\right)-\tilde{\gamma }\left(s,a;s^{\prime }\right)\right)}_{s^{\prime }\in 𝓢,a\in 𝒜}\right)\;.$$ (13)

The sampled internal world model $\tilde{\gamma }(s,a;s^{\prime })$ is pruned in accordance with the estimated support set 𝓢. For the remaining data, we counted the joint occurrences of *s*, *a*, *s*′ and filled the matrix $\tilde{\gamma }(s,a;s^{\prime })$ (pairs (*s*, *a*) index the rows and *s*′ indexes the column). Each non-zero row is normalized to produce a probability distribution and the other rows are discarded. The resulting estimated value for the embodied behavior dimension is *d* ^𝓢,𝓐^ = 3. This means that there is a 3-dimensional face of the polytope $\Delta_{𝒜}^{𝓢}$ of policies on 𝓢, which contains a policy that generates the target behavior. This implies that the target behavior can be generated by a mixture of 4 deterministic policies. The mixture reflects the stochasticity of the system, which results mainly from the discretization of sensor values.

Resulting estimation of the model complexity: It follows that the CRBM is able to represent the target behavior whenever the number of hidden units satisfies

$$m\geq \left|𝓢\right|+d^{𝓢,𝓐}-1=63+3-1=65\;.$$ (14)

To evaluate the tightness of this bound, we conducted a series of experiments, which are explained in the following section.

#### Experiments to evaluate the tightness of the complexity estimation

**Fig 9:**
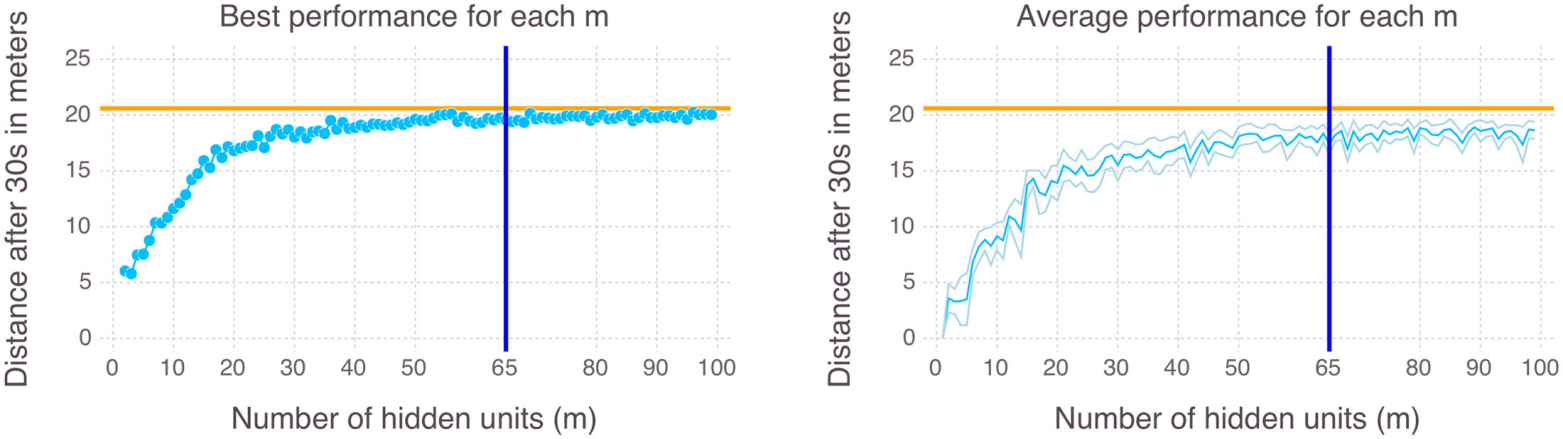
Experimental results. Performance of the best CRBM for different complexity parameters *m* in comparison to the performance of the target behavior (horizontal orange line). The vertical blue line indicates the *m* estimated from the data (see supporting information S2 Text).

Before the experiments can be described, there is an important note to make. This work is concerned with the minimal complexity that is sufficient for controlling a set of desirable behaviors (this set may consist of all possible behaviors or of just one specific behavior). Here, we are *not* concerned with the question how these CRBMs should be trained optimally. This is why we used a standard training algorithm for RBMs [30, 31] and conducted a large scan over different complexity parameters *m*. For each *m* = 1, 2, 3, …, 100 we trained 100 CRBMs with the following learning parameters: epochs = 20000, batch size = 50, learning rate *α* = 1.0, momentum = 0.1, Gaussian distributed noise on sensor data = 0.01, weight cost = 0.001, CRBM Gibbs updates for sampling = 10, on a data set of 10^4^ pairs of sensor and actuator values. Each trained CRBM was evaluated ten times, by applying it to the hexapod and recording the distance covered in 30 seconds. The performance of the CRBMs is measured against the target tripod walking gait, which achieves 20.6 meters during the same time. As we are concerned with the performance that is in principle possible for a given *m*, we choose the policy that covered the most distance at one of the 10 trials (out of the 100 trained policies, for each *m*). The plot in Fig 9 (left-hand side) shows the best performance of the best policy for all scanned values of *m*. The plot in Fig 9 (right-hand side) shows the average performance of the best policy and the standard deviation, over 10 different evaluations, for all values of *m*. The results show that our estimation is fairly tight, which means the performance of the CRBMs converges to the optimal behavior close to the estimated value of *m* = 65. The supporting information S1–S4 Videos show the performance of the best CRBM for *m* = 5, 15, 65, 75.

## Discussion

We presented an approach for studying and implementing cheap design in the context of embodied artificial intelligence. In this context, we referred to cheap design as the reduction of the controller complexity that is possible through an exploitation of the agent’s body and environment. We developed a theory to determine the minimal controller complexity that is sufficient to generate a given set of desired behaviors. Being more precise, we studied the way in which embodiment constraints induce equivalent policies in the sense that they generate the same observable behaviors. This led to the definition of the effective dimension of an embodied system, the *embodied behavior dimension*. In this way, we were able to define low-dimensional policy models that can generate all possible behaviors. Such policy models are related to the classical notion of universal approximation. 

We used CRBMs as a platform of study, for which we presented non-trivial universal approximation results in both the non-embodied and the embodied settings. While the non-embodied universal approximation requires an enormous number of hidden units (exponentially many in the number of input and output units), embodied universal approximation can be achieved using a much smaller number, depending on the effective dimension of the system. Experiments conducted on a walking machine demonstrate the tightness of the estimated number of hidden units for a CRBM controller. This shows the practical utility of our theoretical analysis for embodied artificial intelligence. To the best of our knowledge, the presented formalism and results are amongst the first quantitative contributions to cheap design.

In artificial intelligence, learning is one of the central fields of interest. Crucial for the success of any learning method is the complexity of the underlying model, e.g. a neural network. If the model is chosen too complex, the learning algorithm will likely require too much time and get stuck in a suboptimal solution. If it is chosen too simple, it might not be able to solve the problem at all. This paper deals with the design of concise controller structures with main focus on the model class of conditional restricted Boltzmann machines. In this context, we find that the number *m* of hidden nodes naturally reflects the idea of a cheap or concise control. On the other hand, the total number *m*(*k* + *n* + 1) + *n* of parameters of the conditional restricted Boltzmann machine, increases linearly in *m*, which suggests that a concise control is not only beneficial in terms of mass and energy consumption but also in terms of the quality of learning. It is well known from statistical learning theory [50] that the number of parameters is not always the right measure for controlling the quality of learning. However, for the purpose of this discussion it is sufficient to take an intuitive perspective and interpret low-dimensional models as being beneficial for learning. A central problem within learning theory is the problem of finding the right model complexity for optimal generalization properties. Regularization theory and statistical learning theory provide the right theoretical settings for optimizing the generalization ability of a learning system [50, 51]. Here, an increasing sequence of model complexities is considered, depending on the available data at each time, so that universal approximation is achieved only in the limit of infinite data. The choice of the right model is dynamically adjusted to the available data. In our context, however, the choice of the model is fixed and based on the embodiment of the system. More precisely, we can choose a low-dimensional model, depending on the embodiment dimension, which is our main observation. Whether or not the replacement of a universal approximator by an embodied universal approximator solves the problem of generalization remains an open problem. In any case, the generalization abilities of embodied systems have to be further studied, where concepts from regularization theory and statistical learning theory, such as the structural risk minimization principle [50], will be helpful. In this regard, the freezing-freeing concept [52] seems to point in a similar direction.

Finally, we would like to comment on the applicability of our work to biological systems. The general field of embodied intelligence emerged from the observation that natural intelligent systems tend to incorporate and exploit their morphological properties for the generation of behavior. This suggests the hypothesis that brains of naturally evolved systems are optimized towards concise architectures, referred to as cheap design. Given a fully developed theory of embodied intelligence, this hypothesis should be testable for various biological systems. Although our approach is guided by this long-term vision, in its current form it is not directly applicable to biological systems. In order to approach applicability, it is important to develop methods for efficiently estimating *β* and *α* from biological data. Furthermore, conditional restricted Boltzmann machines represent very simple and unrealistic brain models. Therefore, our results have to be extended to more realistic brain models. Our work may be seen as a guideline for extensions towards understanding cheap design in biological systems.

## Supporting Information

S1 Text Technical proofs. (PDF)

S2 Text Estimation of the Embodied Behavior Dimension based on the Internal World Model. (PDF)

S3 Text Generalizations. (PDF)

S1 FigIllustration of the policy-behavior map.This figure shows an example with ∣𝒮∣ = 3, ∣𝒜∣ = 2, ∣𝒲∣ = 3, and a sensor kernel *β* of affine rank two. Both the policy polytope $\Delta_{𝒜}^{𝒮}$ and the set $\Delta_{𝒜}^{𝒲}$ are three-dimensional cubes. The set $\beta \Delta_{𝒜}^{𝒮}$ of kernels *p*(*w*; *a*) = ∑_*s*_ *β*(*w*; *s*)*π*(*s*; *a*), $\pi \in \Delta_{𝒜}^{𝒮}$, is a two-dimensional polygon (the blue hexagon). This projection by *β* represents one part of the policy-behavior map. The union of all two-dimensional faces of the policy polytope (one of them highlighted in dashed yellow) has the same image as the entire policy polytope.(TIF)

S2 Fig Special causal structure of the sensorimotor loop. The dashed arrows are the ones that we omit within our assumptions.
(TIF)

S3 Fig Causal structure of a SML with internal state. Here *W* ^*t*^, *S* ^*t*^, *C* ^*t*^, *A* ^*t*^ are the states of the world, sensors, internal variable, and actuators at the discrete time *t*.
(TIF)

S1 Video Walking hexapod with *m* = 5. For the indicated value of *m*, 100 CRBMs were trained and the best one was chosen for presentation here. The right-hand side shows the walking behavior of two hexapods, of which one is opaque, while the second one is transparent. The transparent hexapod is controlled by the open-loop sinusoidal controller and displays the target behavior that was used to train the CRBMs. The behavior of the trained CRBM is shown in form of the opaque hexapod. We chose to include both behaviors in the video so that performance of the trained CRBM can be directly compared with the target behavior. The left-hand side in each video shows the internals of the CRBM. From top to bottom: The six squares with the moving blue lines show the raw sensor values for each leg, i.e., the angular values for the knee and shoulder joint over a period of 10 time steps (one second). Below, the activations of the CRBM neurons are shown in the following order (from top to bottom): input layer, hidden layer, output layer. A white box refers to an activation value of 1, while black refers to an activation of 0. The left-hand side is complete with the lower six squares which show how the binary output units translate to motor commands. The orange lines in each square on to the bottom show the motor commands for the knee and shoulder joint of one leg for 10 time steps (1 second).
(MOV)

S2 Video Walking hexapod with *m* = 15. This video has the same layout as S1 Video.
(MOV)

S3 Video Walking hexapod with *m* = 65. This video has the same layout as S1 Video.
(MOV)

S4 Video Walking hexapod with *m* = 75. This video has the same layout as S1 Video.
(MOV)

## References

[1] Pfeifer R, Scheier C (2001) Understanding Intelligence. Cambridge, MA, USA: MIT Press.

[2] Pfeifer R, Bongard JC (2006) How the Body Shapes the Way We Think: A New View of Intelligence. Cambridge, MA: The MIT Press (Bradford Books).

[3] Braitenberg V (1984) Vehicles. Cambridge MA: MIT Press.

[4] Clark DD, Sokoloff L (1999) Circulation and energy metabolism of the brain In: Siegel GJ, Agranoff BW, Albers RW, Fisher SK, Uhler MD, editors, Basic Neurochemistry: Molecular, Cellular and Medical Aspects, Philadelphia: Lippincott-Raven, chapter 31 6th edition, pp. 637–669.

[5] Sokoloff L, Mangold R, Wechsler R, Kennedy C, Kety S (1955) Effect of mental arithmetic on cerebral circulation and metabolism. J Clin Invest 34: 1101–1108. 10.1172/JCI103159 14392225 PMC438861

[6] Sol D, Garcia N, Iwaniuk A, Davis K, Meade A, et al (2010) Evolutionary divergence in brain size between migratory and resident birds. PLoS ONE 5: e9617 10.1371/journal.pone.0009617 20224776 PMC2835749

[7] Pfeifer R (1995) Cognition perspectives from autonomous agents In: Steels L, editor, The Biology and Technology of Intelligent Autonomous Agents, Springer Berlin Heidelberg, volume 144 of *NATO ASI Series* pp. 128–164.

[8] Paul C (2006) Morphological computation: A basis for the analysis of morphology and control requirements. Robotics and Autonomous Systems 54: 619–630.

[9] McGeer T (1990) Passive dynamic walking. International Journal of Robotic Research 9: 62–82. 10.1177/027836499000900206

[10] Hauser H, Ijspeert A, Fchslin R, Pfeifer R, Maass W (2011) Towards a theoretical foundation for morphological computation with compliant bodies. Biological Cybernetics 105: 355–370. 10.1007/s00422-012-0471-0 22290137

[11] Rückert EA, Neumann G (2012) Stochastic optimal control methods for investigating the power of morphological computation. Artificial Life 19: 115–131. 23186345 10.1162/ARTL_a_00085

[12] Polani D (2011) An informational perspective on how the embodiment can relieve cognitive burden. In: Proc. IEEE Symposium Series in Computational Intelligence 2011—Symposium on Artificial Life. IEEE, pp. 78–85.

[13] Zahedi K, Ay N (2013) Quantifying morphological computation. Entropy 15: 1887–1915. 10.3390/e15051887

[14] Haeufle DFB, Günther M, Wunner G, Schmitt S (2014) Quantifying control effort of biological and technical movements: An information-entropy-based approach. Phys Rev E 89: 012716 10.1103/PhysRevE.89.012716 24580266

[15] Ghazi-Zahedi K, Rauh J (2015) Quantifying morphological computation based on an information decomposition of the sensorimotor loop. In: Proceedings of the 13th European Conference on Artificial Life (ECAL 2015), pp. 70–77.

[16] Auerbach JE, Bongard JC (2014) Environmental influence on the evolution of morphological complexity in machines. PLoS Comput Biol 10: e1003399 10.1371/journal.pcbi.1003399 24391483 PMC3879106

[17] Brooks RA (1991) Intelligence without reason. In: Myopoulos J, Reiter R, editors, Proceedings of the 12th International Joint Conference on Artificial Intelligence (IJCAI-91). Sydney, Australia: Morgan Kaufmann publishers Inc.: San Mateo, CA, USA, pp. 569–595.

[18] Brooks RA (1991) Intelligence without representation. Artificial Intelligence 47: 139–159. 10.1016/0004-3702(91)90053-M

[19] Lungarella M, Sporns O (2006) Mapping information flow in sensorimotor networks. PLoS Comput Biol 10. 10.1371/journal.pcbi.0020144 PMC1626158 17069456

[20] Pfeifer R, Lungarella M, Iida F (2007) Self-organization, embodiment, and biologically inspired robotics. Science 318: 1088–1093. 10.1126/science.1145803 18006736

[21] Klyubin AS, Polani D, Nehaniv CL (2004) Tracking information flow through the environment: Simple cases of stigmerg. In: Pollack J, editor, Artificial Life IX: Proceedings of the Ninth International Conference on the Simulation and Synthesis of Living Systems. MIT Press, pp. 563–568.

[22] Ay N, Zahedi K (2014) On the causal structure of the sensorimotor loop In: Prokopenko M, editor, Guided Self-Organization: Inception, Springer pp. 261–294.

[23] Smolensky P (1986) Information processing in dynamical systems: foundations of harmony theory. In: Symposium on Parallel and Distributed Processing. pp. 194–281.

[24] Freund Y, Haussler D (1994) Unsupervised Learning of Distributions of Binary Vectors Using Two Layer Networks. Technical report. Computer Research Laboratory, University of California, Santa Cruz.

[25] Larochelle H, Bengio Y (2008) Classification using discriminative restricted Boltzmann machines. In: Cohen WW, McCallum A, Roweis ST, editors, Proceedings of the 25th International Conference on Machine Learning (ICML 2008). volume 307, pp. 536–543.

[26] Salakhutdinov R, Mnih A, Hinton GE (2007) Restricted Boltzmann machines for collaborative filtering. In: Proceedings of the 24th International Conference on Machine Learning (ICML 2007). pp. 791–798.

[27] Sutskever I, Hinton GE (2007) Learning multilevel distributed representations for high-dimensional sequences. Proceeding of the 11th International Conference on Artificial Intelligence and Statistics: 548–555.

[28] Taylor GW, Hinton GE, Roweis S (2007) Modeling human motion using binary latent variables In: NIPS 19 MIT Press, pp. 1345–1352.

[29] Sallans B, Hinton GE (2004) Reinforcement learning with factored states and actions. J Mach Learn Res 5: 1063–1088.

[30] Hinton GE (2002) Training products of experts by minimizing contrastive divergence. Neural Computation 14: 1771–1800. 10.1162/089976602760128018 12180402

[31] Hinton GE (2012) A practical guide to training restricted Boltzmann machines In: Montavon G, Orr G, Müller KR, editors, Neural Networks: Tricks of the Trade, Springer Berlin Heidelberg, volume 7700 of *Lecture Notes in Computer Science* pp. 599–619.

[32] Bengio Y (2009) Learning deep architectures for AI. Foundations and Trends in Machine Learning 2: 1–127. 10.1561/2200000006

[33] Long PM, Servedio RA (2010) Restricted Boltzmann machines are hard to approximately evaluate or simulate. In: Fürnkranz J, Joachims T, editors, Proceedings of the 27th International Conference on Machine Learning (ICML 2010). Omnipress, pp. 703–710.

[34] Le Roux N, Bengio Y (2008) Representational power of restricted Boltzmann machines and deep belief networks. Neural Computation 20: 1631–1649. 10.1162/neco.2008.04-07-510 18254699

[35] Montúfar G, Ay N (2011) Refinements of universal approximation results for deep belief networks and restricted Boltzmann machines. Neural Computation 23: 1306–1319. 10.1162/NECO_a_00113 21299421

[36] Montúfar G, Rauh J, Ay N (2011) Expressive power and approximation errors of restricted Boltzmann machines In: Shawe-Taylor J, Zemel R, Bartlett P, Pereira F, Weinberger K, editors, NIPS 24 pp. 415–423.

[37] Martens J, Chattopadhya A, Pitassi T, Zemel R (2013) On the expressive power of restricted Boltzmann machines In: Burges C, Bottou L, Welling M, Ghahramani Z, Weinberger K, editors, NIPS 26 pp. 2877–2885.

[38] Montúfar G, Ay N, Ghazi-Zahedi K (2014) Expressive power of conditional restricted Boltzmann machines. To appear in J Mach Learn Res. arXiv preprint arXiv:14023346.

[39] Shannon CE (2001) A mathematical theory of communication. SIGMOBILE Mob Comput Commun Rev 5: 3–55. 10.1145/584091.584093

[40] Bauer H (1996) Probability Theory. De Gruyter studies in mathematics. Bod Third Party Titles.

[41] Pearl J (2009) Causality: Models, Reasoning and Inference. New York, NY, USA: Cambridge University Press, 2nd edition.

[42] Aström K, Murray R (2010) Feedback Systems: An Introduction for Scientists and Engineers. Princeton University Press.

[43] Rivoire O, Leibler S (2011) The value of information for populations in varying environments. Journal of Statistical Physics 142: 1124–1166. 10.1007/s10955-011-0166-2

[44] Bialek W, Nemenman I, Tishby N (2001) Predictability, complexity, and learning. Neural Computation 13: 2409–2463. 10.1162/089976601753195969 11674845

[45] Zahedi K, Ay N, Der R (2010) Higher coordination with less control—a result of informaion maximiation in the sensorimotor loop. Adaptive Behaviour 18: 338–355. 10.1177/1059712310375314

[46] Ay N, Montúfar G, Rauh J (2013) Selection criteria for neuromanifolds of stochastic dynamics In: Yamaguchi Y, editor, Advances in Cognitive Neurodynamics (III), Springer pp. 147–154.

[47] Brooks RA (1989) A robot that walks; emergent behaviors from a carefully evolved network. Neural Comput 1: 253–262. 10.1162/neco.1989.1.2.253

[48] Zahedi K, von Twickel A, Pasemann F (2008) Yars: A physical 3d simulator for evolving controllers for real robots In: Carpin S, Noda I, Pagello E, Reggiani M, von Stryk O, editors, SIMPAR 2008. Springer, LNAI 5325, pp. 71–82.

[49] Coumans E (2012). Bullet physic sdk manual. www.bulletphysics.org.

[50] Vapnik VN (1998) Statistical Learning Theory. Wiley-Interscience.

[51] Bishop CM (2006) Pattern Recognition and Machine Learning (Information Science and Statistics). Secaucus, NJ, USA: Springer-Verlag New York, Inc.

[52] Berthouze L, Lungarella M (2004) Motor skill acquisition under environmental perturbations: On the necessity of alternate freezing and freeing of degrees of freedom. Adaptive behavior 12: 47–64. 10.1177/105971230401200104

