# Supplementary material for: A Theory of Cheap Control in Embodied Systems
Source: PLoS Comput Biol. 2015 Sep 1;11(9):e1004427. doi: 10.1371/journal.pcbi.1004427 (PMC4556690; doi:10.1371/journal.pcbi.1004427)
Supplement: S1 Text — (PDF) [file pcbi.1004427.s001.pdf]

S1 Text. Technical Proofs

Details of Eq. (10). The set $\mathfrak{B} = \psi(\Delta^{\mathscr{S}}_{\mathscr{A}})$ is a polytope, because $\Delta^{\mathscr{S}}_{\mathscr{A}}$ is a polytope and ψ is an affine map. The dimension $d = \dim(\mathfrak{B})$ is equal to the number of affinely independent vectors in $\mathfrak{B}$. That is, d is the dimension of the vector space $\mathrm{aff}(\mathfrak{B}) := \{\lambda_1 p_1 + \cdots + \lambda_N p_N \colon p_1, \dots, p_N \in \mathfrak{B}, \lambda_1, \dots, \lambda_N \in \mathbb{R}, \lambda_1 + \cdots + \lambda_N = 0, N \in \mathbb{N}\}$.

Now, note that $\mathrm{aff}(\mathfrak{B}) = \mathrm{aff}(\psi(\Delta^{\mathscr{S}}_{\mathscr{A}})) = \psi(\mathrm{aff}(\Delta^{\mathscr{S}}_{\mathscr{A}}))$. Hence $\mathrm{aff}(\mathfrak{B})$ is spanned by the image (by ψ) of a basis of $\mathrm{aff}(\Delta^{\mathscr{S}}_{\mathscr{A}})$. A basis of $\mathrm{aff}(\Delta^{\mathscr{S}}_{\mathscr{A}})$ can be obtained by building differences of vertices of $\Delta^{\mathscr{S}}_{\mathscr{A}}$, as follows. The vertices of $\Delta^{\mathscr{S}}_{\mathscr{A}}$ are the deterministic policies $\pi^f(s;a) := \delta_{f(s)}(a)$, for all $s \in \mathscr{S}$ and $a \in \mathscr{A}$, each of which is characterized by a function $f \colon \mathscr{S} \to \mathscr{A}$. We fix a deterministic policy π^f, with $f(s) = a_0$ for all s, for some $a_0 \in \mathscr{A}$, and consider the differences $e_{(s,a)} := \pi^f - \pi^{f_{(s,a)}}$ for all possible pairs (s, a) with $a \neq a_0$, where $f_{(s,a)}$ is the function that differs from f only at s, taking value $f_{(s,a)}(s) = a$. This set of $e_{(s,a)}$'s is a basis of $\mathrm{aff}(\Delta^{\mathscr{S}}_{\mathscr{A}})$. There are $|\mathscr{S}|(|\mathscr{A}| - 1)$ of these vectors, which corresponds with the dimension of $\Delta^{\mathscr{S}}_{\mathscr{A}}$.

By the above discussion, the vector space $\mathrm{aff}(\mathfrak{B})$ is spanned by the image

$$p_{(s,a)}(w; dw) := \psi(e_{(s,a)})(w; dw') = \beta(w; s)(\alpha(w, a_0; dw') - \alpha(w, a; dw'))$$

of the basis vectors $e_{(s,a)}$, for all possible pairs (s, a) with $a \neq a_0$. Therefore, the dimension $d = \dim(\mathfrak{B}) = \dim(\mathrm{aff}(\mathfrak{B}))$ is equal to the number of linearly independent $p_{(s,a)}$'s. □

Details of the exponential family from Eq. (11). We consider an exponential family $\mathcal{E}$ of probability distributions on the set $\mathscr{A}^{\mathscr{S}}$ of functions $f \colon \mathscr{S} \to \mathscr{A}$. Let this exponential family be specified by the sufficient statistic $F := \psi \circ \eta$, where $\eta \colon \mathscr{A}^{\mathscr{S}} \to \Delta^{\mathscr{S}}_{\mathscr{A}};\ f \mapsto \pi^f$, $\pi^f(s;a) := \delta_{f(s)}(a)$ for all $s \in \mathscr{S}$ and $a \in \mathscr{A}$, and ψ is the policy-behavior map, represented by the matrix $E \in \mathbb{R}^{d \times (\mathscr{S} \times \mathscr{A})}$. Note that, given a basis of $\mathrm{aff}(\psi(\Delta^{\mathscr{S}}_{\mathscr{A}}))$, composed of d vectors in $\mathrm{aff}(\psi(\Delta^{\mathscr{S}}_{\mathscr{A}}))$, we can represent each $e_{(s,a)}$ and $\psi(\pi)$ with respect to this basis by a vector of length $d \leq |\mathscr{S}|(|\mathscr{A}| - 1)$. The exponential family $\mathcal{E}$ consists of all probability distributions of the form

$$p_\theta(f) = \frac{\exp(\theta^\top F(f))}{\sum_{f'} \exp(\theta^\top F(f'))}, \quad f \in \mathscr{A}^{\mathscr{S}}, \text{ for all } \theta \in \mathbb{R}^d.$$

The *moment map* μ maps probability distributions to the corresponding expectation value of the sufficient statistics,

$$\mu \colon \Delta_{\mathscr{A}^{\mathscr{S}}} \to \mathbb{R}^d;\ p \mapsto \sum_f F(f)p(f) = \sum_f \psi(\eta(f))p(f) = \psi(\sum_f \eta(f)p(f)) = \psi(\pi^p),$$

where $\pi^p := \eta(p) = \sum_f \pi^f p(f) \in \Delta^{\mathscr{S}}_{\mathscr{A}}$. A key property of the moment map is that it maps the closure $\overline{\mathcal{E}}$ of $\mathcal{E}$ bijectively to the set $\mu(\Delta_{\mathscr{A}^{\mathscr{S}}})$ of all possible expectation values. We have

$$\psi(\eta(\overline{\mathcal{E}})) = \mu(\overline{\mathcal{E}}) = \mu(\Delta_{\mathscr{A}^{\mathscr{S}}}) = \psi(\Delta^{\mathscr{S}}_{\mathscr{A}}).$$

Now we only need to show that the set $\eta(\mathcal{E}) = \{\pi^p = \sum_f \pi^f p(f) \colon p \in \mathcal{E}\}$ is contained in $\mathcal{E}^{\mathscr{S}}_{\mathscr{A}}$. That this is true can be seen from

$$\begin{aligned}\pi^{p_\theta}(s;a) =& \sum_f \pi^f(s;a)p_\theta(f) = \sum_{f \colon f(s)=a} p_\theta(f) = p_\theta(\{f \colon f(s) = a\}) = \frac{\exp(\theta^\top E(s,a))}{\sum_{a'} \exp(\theta^\top E(s,a'))} \\ =& \pi_\theta(s;a), \quad a \in \mathscr{A}, s \in \mathscr{S}, \quad \text{for all } \theta \in \mathbb{R}^d,\end{aligned}$$

where we used

$$p_\theta(f) = \frac{\exp(\theta^\top E(\pi^f))}{\sum_{f'} \exp(\theta^\top E(\pi^{f'}))} = \frac{\exp(\theta^\top \sum_s E(s, f(s)))}{\sum_{f'} \exp(\theta^\top \sum_{s'} E(s', f'(s')))} = \prod_s \frac{\exp(\theta^\top E(s, f(s)))}{\sum_a \exp(\theta^\top E(s, a))}$$
$$= \prod_s p_\theta(\{f' : f'(s) = f(s)\}).$$

In fact, since μ is a bijection between $\overline{\mathcal{E}}$ and $\mu(\Delta_{\mathscr{A}^{\mathscr{S}}}) = \psi(\Delta_{\mathscr{A}}^{\mathscr{S}})$, we have that ψ is a bijection between $\overline{\mathcal{E}_{\mathscr{A}}^{\mathscr{S}}} = \eta(\overline{\mathcal{E}})$ and $\psi(\Delta_{\mathscr{A}}^{\mathscr{S}})$. □

Proof of Lemma 1. Assume first that $\mathcal{S} = \mathscr{S}$. The policy-behavior map ψ is a linear map that projects the policy polytope $\Delta_{\mathscr{A}}^{\mathscr{S}}$ into a polytope $\mathfrak{B} \subseteq \Delta_{\mathscr{W}}^{\mathscr{W}}$ of dimension d. Furthermore, a d-dimensional linear projection of a polytope is equal to the projection of the union of its d-dimensional faces. See the supporting information S1 Fig for an illustration of what we mean. This implies that the behavior generated by a given policy from $\Delta_{\mathscr{A}}^{\mathscr{S}}$ can also be generated by a policy from a d-dimensional face of $\Delta_{\mathscr{A}}^{\mathscr{S}}$.

In addition, the d-dimensional faces of the policy polytope $\Delta_{\mathscr{A}}^{\mathscr{S}}$ consist of those policies with at most $|\mathscr{S}| + d$ non-zero entries. The arguments for this are as follows. The policy polytope is a product of simplices $\Delta_{\mathscr{A}}^{\mathscr{S}} = \times_{s\in\mathscr{S}} \Delta_{\mathscr{A}}$, where the s-th factor corresponds to the set of all possible probability distributions $\pi(s;\cdot)$. The faces of $\Delta_{\mathscr{A}}^{\mathscr{S}}$ are products of faces of its factors and have the form $\times_{s\in\mathscr{S}} \Delta_{\mathscr{A}_s}$, where $\mathscr{A}_s \subseteq \mathscr{A}$ for all $s \in \mathscr{S}$. Each face of $\Delta_{\mathscr{A}}^{\mathscr{S}}$ corresponds to a choice of positions $\mathscr{A}_s \subseteq \mathscr{A}$ of the non-zero entries of $\pi(s;\cdot)$ for all $s \in \mathscr{S}$. The d-dimensional faces are those for which $\sum_{s\in\mathscr{S}}(|\mathscr{A}_s| - 1) = d$, meaning that they consist of policies which have at most $\sum_{s\in\mathscr{S}} |\mathscr{A}_s| = |\mathscr{S}| + d$ non-zero entries.

Consider now $\mathcal{S} \subseteq \mathscr{S}$ and $\mathcal{A}_s, s \in \mathcal{S}$. To understand this case, we consider the map which first projects $\Delta_{\mathscr{A}}^{\mathscr{S}}$ to $\times_{s\in\mathcal{S}}\Delta_{\mathcal{A}_s}$ and then projects $\times_{s\in\mathcal{S}}\Delta_{\mathcal{A}_s}$ by the restriction of ψ to $\times_{s\in\mathcal{S}}\mathcal{A}_s$. We can use the same arguments as above, with the difference that now only need to represent the $d^{\mathcal{S},\mathcal{A}}$-dimensional faces of the polytope $\times_{s\in\mathcal{S}}\Delta_{\mathcal{A}_s}$. □
