# Supplementary material for: A Theory of Cheap Control in Embodied Systems
Source: PLoS Comput Biol. 2015 Sep 1;11(9):e1004427. doi: 10.1371/journal.pcbi.1004427 (PMC4556690; doi:10.1371/journal.pcbi.1004427)
Supplement: S2 Text — (PDF) [file pcbi.1004427.s002.pdf]

S2 Text. Estimation of the Embodied Behavior Dimension based on the Internal World Model

In many situations, the embodied behavior dimension is not available from a perspective that is intrinsic to the agent, as the agent does not have direct access to the sensor kernel β nor to the world kernel α. From that perspective, only an internal version of the world model is accessible, which we refer to as *internal world model.* It is defined as a kernel $\gamma \in \Delta_{\mathscr{S}}^{\mathscr{S}\times\mathscr{A}}$, assigning to each sensor state s with positive probability and each actuator state a the next sensor state s', that is,

$$\gamma(s,a;s') = \int_{\mathscr{W}} \left\{ \int_{\mathscr{W}} \beta(w';s')\, \alpha(w,a;dw') \right\} \mathbb{P}(s;dw)\,,$$

where

$$\mathbb{P}(s;dw) \;:=\; \frac{\beta(w;s)}{\int_{\mathscr{W}} \beta(w'';s)\,\mathbb{P}(dw'')}\,\mathbb{P}(dw)\,.$$

Note that the internal world model is not completely determined by β and α. It also depends on the distribution $\mathbb{P}(dw)$ of the world states w. If we choose this distribution to be a fixed reference distribution of world states, then the world model will be determined by α and β only. However, in order to describe the actual distribution of world states, we have to take into account the contribution of the agent's policy π. This implies that, if the policy is subject to changes in terms of a learning process, then, in general, the world model will also be time dependent.

On the other hand, γ is the only information about world dynamics that is intrinsically available to the agent. The extent to which γ is not a good replacement for α depends on how much the agent can "see" from the world with its sensors. If the agent has direct access to the world state, that is $W^t = S^t$, then α and γ coincide. However, this is not very realistic. Generically, only partial observation of the world is possible. Now the question arises whether it is possible to determine the embodied behavior dimension d in terms of γ even in cases where the agent has only partial access to the world state. This is indeed possible under specific conditions which are satisfied in our experimental setup. We first present these conditions in general terms, before we then relate them to our experiment at the end of this section. Let the world state w consist of two parts s and r, where s is directly accessible to the agent and r is the remaining part of the world, which is hidden to the agent. The situation is illustrated in the supporting information S2 Fig.

In this interpretation of the world state, the sensor kernel β is simply the identity map $s \mapsto s$. Furthermore, this interpretation sets structural constraints on the world transition kernel α, which assigns a probability distribution of the next world state $w' = (s', r')$ given the current world state $w = (s, r)$ and an actuator value a. As r is assumed to be hidden to the agent, s' should not depend on r. This leads to the following natural factorization of α:

$$\alpha(s,r,a;s',dr') \;=\; \alpha^S(s,a;s') \cdot \alpha^R(r,s,s';dr')\,.$$

With this assumption, we obtain as internal world model

$$\gamma(s,a;s') \;=\; \alpha^S(s,a;s'), \qquad \text{whenever } \mathbb{P}(s) > 0\,,$$

and the following transition probabilities from a world state $w = (s, r)$ with positive probability to a world

states $w' = (s', r')$:

$$\begin{aligned} \mathbb{P}^{\pi}(s, r; s', dr') &= \sum_{s'', a} \beta(s, r; s'')\, \pi(s''; a)\, \alpha(s, r, a; s', dr') \\ &= \sum_{a} \pi(s; a)\, \alpha^{S}(s, a; s')\, \alpha^{R}(r, s, s'; dr') \\ &= \alpha^{R}(r, s, s'; dr') \underbrace{\sum_{a} \pi(s; a)\, \alpha^{S}(s, a; s')}_{=: \mathbb{Q}^{\pi}(s; s')} . \end{aligned}$$

This shows that $\mathbb{P}^{\pi} = \mathbb{P}^{\pi^*}$ if and only if $\mathbb{Q}^{\pi} = \mathbb{Q}^{\pi^*}$, and therefore the embodied behavior dimension is given by the dimension of the image of $\pi \mapsto \mathbb{Q}^{\pi}$.

This applies to our hexapod experiment discussed in section "Experiments with a Hexapod" for the following reason. In the special case of the tripod gait of a hexapod on an even and featureless plane, the next joint angles S^{t+1} are determined only by the current joint angles S^t and the current action A^t. The rest of the world, here denoted by R, contains information such as the contact points of the legs with the ground. This information is carried from one time step to the next, as it determines how the hexapod walks along the plane. Nevertheless, the contact points of the legs do not influence the joint angles. Hence, in our experiment, S' is conditionally independent of R given S and A. Furthermore, R' is conditionally independent of A given R, S, S', as the contact points of the legs with the ground are only determined by the relative joint angles, and not by the current action. Therefore, we can estimate the embodied behavior dimension using the rank of the internal world model.

Next we describe how to compute the embodied behavior dimension. Consider each policy π as a matrix with rows indexed by s and columns indexed by a. Then the s-th row $\mathbb{Q}^{\pi}(s; \cdot)$ is the image of the s-th row $\pi(s; \cdot)$ by the matrix $(\alpha^{S}(s, a; s'))_{s' \in \mathscr{S}, a \in \mathscr{A}}$. We have one individual map like this for each $s \in \mathscr{S}$. The dimension of the image of the s-th map is given by the rank of the matrix $(\alpha^{S}(s, a_0; s') - \alpha^{S}(s, a; s'))_{s' \in \mathscr{S}, a \in \mathscr{A}}$. Here a_0 is an arbitrary fixed value in $\mathscr{A}$. By taking this difference matrix we account for the fact that the entries in the s-th row of the policy are not independent but satisfy $\sum_a \pi(s; a) = 1$. As discussed above, $\gamma(s, a; s') = \alpha^{S}(s, a; s')$, whenever $\mathbb{P}(s) > 0$. Since the individual maps of rows are independent for all s, the image dimension of the composite map is the sum of the image dimensions of the individual maps, and

$$d = \sum_{s \in \mathscr{S}} \operatorname{rank}((\gamma(s, a_0; s') - \gamma(s, a; s'))_{s' \in \mathscr{S}, a \in \mathscr{A}}),$$

where a_0 is any fixed value in $\mathscr{A}$.

In the implementation we sampled the internal world model for a specific behavior. This results in a sampled internal world model $\tilde{\gamma}$, which may be an incomplete version of γ (for instance, the policy may assign zero probability to some pairs (s, a) and hence deliver no data for the corresponding entries of the sampled internal world model). Nevertheless, the behavior used for sampling the internal world model is contained in some set $\mathcal{B}^{\mathcal{S}, \mathcal{A}}$ (see section "Cheap Representation of Embodied Behaviors"). The set $\mathcal{S}$ can be estimated as discussed in the main text and $\mathcal{A}_s$ as the set of actuator values $a \in \mathscr{A}$ for which the (s, a)-th row $\tilde{\gamma}(s, a; \cdot)$ of the sampled internal world model is non-zero. The important observation at this point is that the policy behavior map estimated from the sampled internal world model $\tilde{\gamma}$ corresponds precisely to the restriction of the policy behavior map to the set $\Delta^{\mathcal{S}}_{\mathcal{A}}$. This always hold, irrespectively of the specific $\mathcal{A}$, which may remain unknown to us. Therefore, the affine rank of the policy behavior map sampled from a behavior in $\mathcal{B}^{\mathcal{S}, \mathcal{A}}$ corresponds to the dimension $d^{\mathcal{S}, \mathcal{A}}$. The estimated value is given by

$$d^{\mathcal{S}, \mathcal{A}} = \sum_{s \in \mathcal{S}} \operatorname{rank}((\tilde{\gamma}(s, a_0; s') - \tilde{\gamma}(s, a; s'))_{s' \in \mathcal{S}, a \in \mathscr{A}}).$$
