# Supplementary material for: A Theory of Cheap Control in Embodied Systems
Source: PLoS Comput Biol. 2015 Sep 1;11(9):e1004427. doi: 10.1371/journal.pcbi.1004427 (PMC4556690; doi:10.1371/journal.pcbi.1004427)
Supplement: S3 Text — (PDF) [file pcbi.1004427.s003.pdf]

S3 Text. Generalizations

SML with Internal State

In the main part of the paper we considered reactive SMLs. In a more general setting, the agent may be equipped with some sort of memory or internal representation of the world. In this case, besides from the world state, the sensor state, and the actuator state, the SML also includes an internal state variable. As in the reactive SML, the dynamics of these variables are governed by Markov transition kernels, but the causality structure is slightly different. Let $\mathscr{W}$, $\mathscr{S}$, $\mathscr{C}$, and $\mathscr{A}$ denote the sets of possible states of the world, the sensors, the internal state, and the actuators. Then the Markov kernels are

$$\begin{aligned} \beta\colon \mathscr{W} &\to \Delta_{\mathscr{S}}\,, \\ \varphi\colon \mathscr{C}\times\mathscr{S} &\to \Delta_{\mathscr{C}}\,, \\ \pi\colon \mathscr{C} &\to \Delta_{\mathscr{A}}\,, \\ \alpha\colon \mathscr{W}\times\mathscr{A} &\to \Delta_{\mathscr{W}}\,. \end{aligned}$$

See the supporting information S3 Fig for an illustration of this causality structure.

As in the reactive case discussed in the main text, here we also want to consider the (extrinsic) behavior of the agent, which is described in terms of the stochastic process of world states. In this case, however, we condition these processes not only on an initial world state but also on an initial internal state. The difficulty arising here is that, in general, in the presence of an internal state the stochastic process over the world states is not Markovian. The world state transition at each time step does not only depend on the previous world state but it also depends on a longer history, encoded in the internal state.

For example, when navigating a territory, a robot endowed with an internal state could operate in the following way. If at a given time step the robot detects an obstacle ahead, $s =$ "obstacle", then, in conjunction with a current internal state $c =$ "safe", the new internal state could become $c' =$ "attentive", in which case the policy would choose $a =$ "maintain direction". However, if the current internal state was $c =$ "attentive", the new internal state could become $c' =$ "alert", in which case the policy would choose between the actions $a' =$ "turn left" and $a' =$ "turn right" with probability $\frac{1}{2}$. This example shows that the internal state may contain information about the history of world and sensor states, which is not available from the current world and sensor states alone.

Nonetheless, for any fixed choice of the kernels $\beta, \varphi, \pi, \alpha$ and a starting value (w^0, c^0) at time $t = 0$, the SML defines a (discrete-time homogeneous) Markov chain with state space $\mathscr{W}\times\mathscr{S}\times\mathscr{C}\times\mathscr{A}$. The transition probabilities of this chain are given by

$$\mathbb{P}^\pi(w^0, s^0, c^0, a^0; dw^1, s^1, c^1, a^1) = \alpha(w^0, a^0; dw^1)\beta(w^1; ds^1)\varphi(c^0, s^1; dc^1)\pi(c^1; da^1)\,.$$

Furthermore, the process with state space $\mathscr{W}\times\mathscr{C}$ is also Markovian. The transition probabilities of this chain are given by

$$\psi(w, c; dw', dc') = \int_{\mathscr{S}'}\int_{\mathscr{A}} \pi(c; da)\alpha(w, a; dw')\beta(w'; ds')\varphi(c, s'; dc')\,.$$

The process on $\mathscr{W}$ (extrinsic behavior) is the marginal of the process on $\mathscr{W}\times\mathscr{C}$ (extrinsic-intrinsic behavior). We can study some properties of the extrinsic behavior in terms of the properties of the extrinsic-intrinsic behavior. The latter is easier to analyze, since it is Markovian. In particular, we can study it in the same way we studied the extrinsic behavior in the reactive SML.

More explicitly we have the following. Writing $\xi(w, c; dw') = \int_{\mathscr{A}} \pi(c; da)\alpha(w, a; dw')$ and $\phi(w', c; dc') = \int_{\mathscr{S}'} \beta(w'; ds')\varphi(c, s'; dc')$, the transition probabilities for the process on $\mathscr{W}\times\mathscr{C}$ are given by

$$\psi(w, c; dw', dc') = \xi(w, c; dw')\phi(w', c; dc')\,.$$

For each (w, c), the probability distribution $\xi(w, c; \cdot) \in \Delta_{\mathscr{W}}$ is the projection of $\pi(c; \cdot) \in \Delta_{\mathscr{A}}$ by the linear map defined by $\alpha(w, \cdot; \cdot)$. If the intersection of the null-spaces of $\alpha(w, \cdot; \cdot)$ for all w has a positive dimension, then there is a positive dimensional set of policies π that are mapped to the same ξ and hence to the same behavior. In order to obtain that behavior, it is sufficient to represent one of the policies that map to ξ, in contrast to the potentially much larger set of all policies that map to the same ξ. A similar observation applies to φ. This shows that already when considering the process on $\mathscr{W} \times \mathscr{C}$ (the combined extrinsic-intrinsic behavior of the agent), many policies may be identified. Embodiment constraints restrict the possible behaviors. When considering only the process over $\mathscr{W}$ (the extrinsic behavior of the agent), many more policies may be identified with the same behavior. The detailed study of projections from combined behaviors to extrinsic behaviors is left for future work.

Continuous Sensor and Actuator State Spaces

We have considered systems where $\mathscr{S}$ and $\mathscr{A}$ are finite sets. In some case it can be more natural to consider continuous sensor and actuator spaces. The continuous case brings some subtleties with it. In particular, the set of policies with continuous state spaces is infinite dimensional. In this case one has to depart from linear algebra and use functional analysis. Furthermore, in the setting of continuous sensor and actuator spaces usually it is not possible to achieve universal approximation by one fixed model. Rather, one says that a class of models has the universal approximation property, meaning that for each given error tolerance, there is a model in that class, that can approximate to within that error tolerance. Nonetheless, one can measure the approximation performance in terms of the (finite) number of parameters or hidden variables that a model needs in order to satisfy a given error tolerance. Continuous policy models can be defined in terms of stochastic feed-forward neural networks with continuous variables or also in terms of CRBMs with Gaussian output units. Here, the complexity of a model can be measured in terms of the number of hidden variables.
