# Supplementary figures and images for: A Theory of Cheap Control in Embodied Systems
Source: PLoS Comput Biol. 2015 Sep 1;11(9):e1004427. doi: 10.1371/journal.pcbi.1004427 (PMC4556690; doi:10.1371/journal.pcbi.1004427)

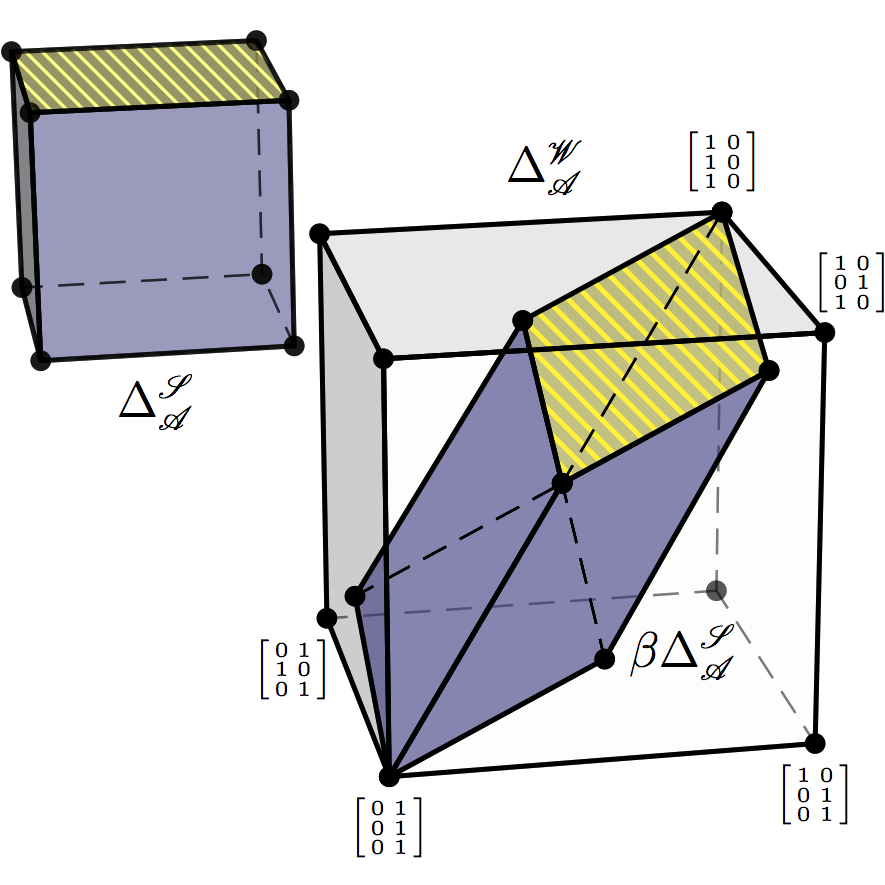

Supplement: S1 Fig — This figure shows an example with ∣𝒮∣ = 3, ∣𝒜∣ = 2, ∣𝒲∣ = 3, and a sensor kernel β of affine rank two. Both the policy polytope Δ𝒜𝒮 and the set Δ𝒜𝒲 are three-dimensional cubes. The set βΔ𝒜𝒮 of kernels p(w; a) = ∑s β(w; s)π(s; a), π∈Δ𝒜𝒮, is a two-dimensional polygon (the blue hexagon). This projection by β represents one part of the policy-behavior map. The union of all two-dimensional faces of the policy polytope (one of them highlighted in dashed yellow) has the same image as the entire policy polytope. (TIF) [file pcbi.1004427.s004.tif]

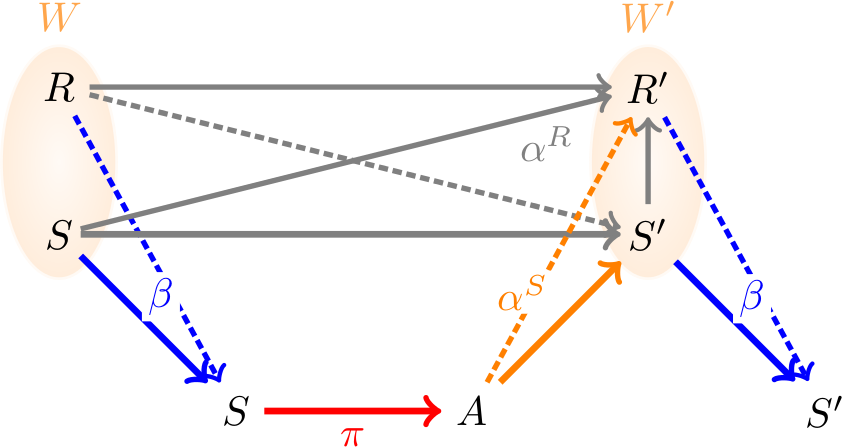

Supplement: S2 Fig — The dashed arrows are the ones that we omit within our assumptions. (TIF) [file pcbi.1004427.s005.tif]

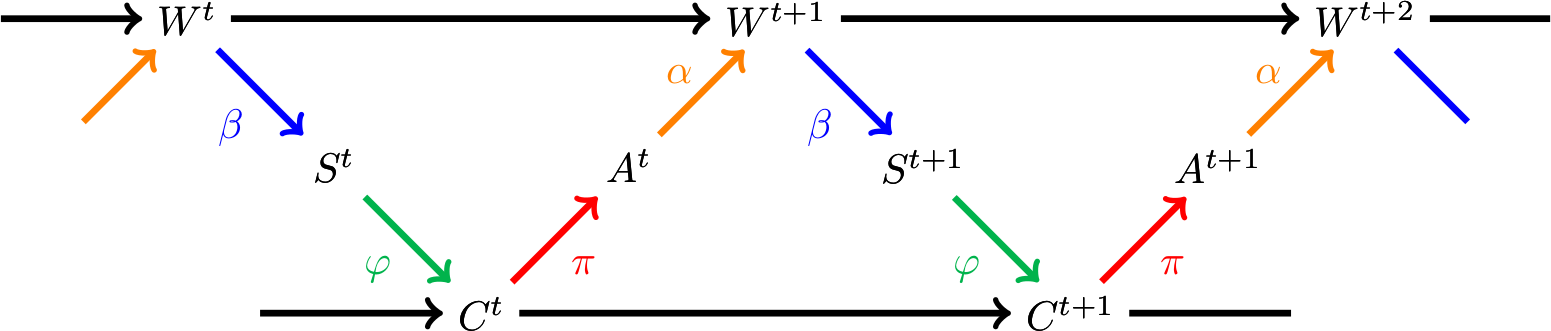

Supplement: S3 Fig — Here W t, S t, C t, A t are the states of the world, sensors, internal variable, and actuators at the discrete time t. (TIF) [file pcbi.1004427.s006.tif]
